# Metabolism characterization and toxicity of *N*-hydap, a marine candidate drug for lung cancer therapy by LC–MS method

**DOI:** 10.1007/s13659-024-00455-x

**Published:** 2024-05-21

**Authors:** Jindi Lu, Weimin Liang, Yiwei Hu, Xi Zhang, Ping Yu, Meiqun Cai, Danni Xie, Qiong Zhou, Xuefeng Zhou, Yonghong Liu, Junfeng Wang, Jiayin Guo, Lan Tang

**Affiliations:** 1 NMPA Key Laboratory for Research and Evaluation of Drug Metabolism, Guangdong Provincial Key Laboratory of New Drug Screening, Guangdong-Hong Kong-Macao Joint Laboratory for New Drug Screening, Southern Medical University Hospital of Integrated Traditional Chinese and Western Medicine, School of Pharmaceutical Sciences, Southern Medical University, Guangzhou, 510515 China; 2 CAS Key Laboratory of Tropical Marine Bio-Resources and Ecology/Guangdong Key Laboratory of Marine Materia Medica, South China Sea Institute of Oceanology, Chinese Academy of Sciences, Guangzhou, 510301 China

**Keywords:** *N*-Hydap, Metabolism, Pharmacokinetics, DMEs, Toxicity, DDIs

## Abstract

*N*-Hydroxyapiosporamide (*N*-hydap), a marine product derived from a sponge-associated fungus, has shown promising inhibitory effects on small cell lung cancer (SCLC). However, there is limited understanding of its metabolic pathways and characteristics. This study explored the in vitro metabolic profiles of *N*-hydap in human recombinant cytochrome P450s (CYPs) and UDP-glucuronosyltransferases (UGTs), as well as human/rat/mice microsomes, and also the pharmacokinetic properties by HPLC–MS/MS. Additionally, the cocktail probe method was used to investigate the potential to create drug-drug interactions (DDIs). *N*-Hydap was metabolically unstable in various microsomes after 1 h, with about 50% and 70% of it being eliminated by CYPs and UGTs, respectively. UGT1A3 was the main enzyme involved in glucuronidation (over 80%), making glucuronide the primary metabolite. With a favorable bioavailability of 24.0%, *N*-hydap exhibited a higher distribution in the lungs (26.26%), accounting for its efficacy against SCLC. Administering *N*-hydap to mice at normal doses via gavage did not result in significant toxicity. Furthermore, *N*-hydap was found to affect the catalytic activity of drug metabolic enzymes (DMEs), particularly increasing the activity of UGT1A3, suggesting potential for DDIs. Understanding the metabolic pathways and properties of *N*-hydap should improve our knowledge of its drug efficacy, toxicity, and potential for DDIs.

**Graphical Abstract:**

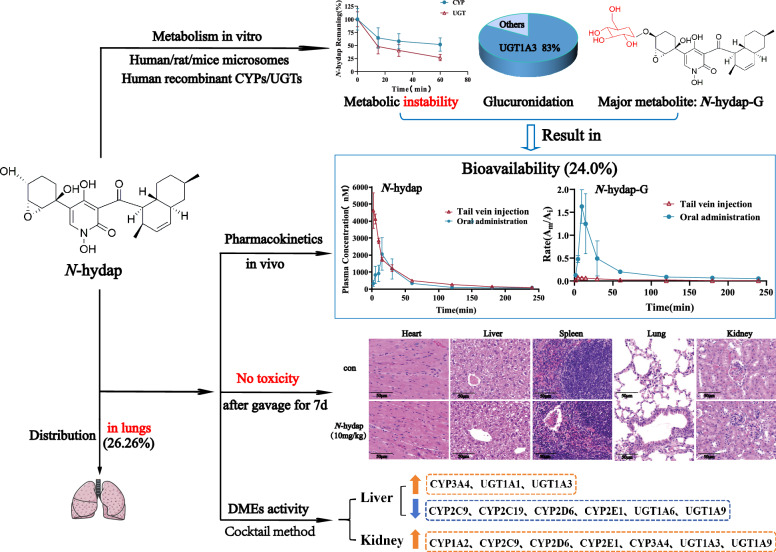

**Supplementary Information:**

The online version contains supplementary material available at 10.1007/s13659-024-00455-x.

## Introduction

SCLC is an aggressive neuroendocrine carcinoma, characterized by rapid proliferation, drug resistance and an unfavorable prognosis, accounting for about 15% of all lung cancer cases and is the leading cause of cancer-related death [1]. Current standard treatments for SCLC: a combination of cisplatin/carboplatin with etoposide, but drug resistance develops rapidly, 5-year survival rate < 20% [2]; 1st-line therapy atezolizumab, comes with health costs of $382,000/quality-adjusted life-year [3]; 2nd-line therapy topotecan, can cause severe bone marrow suppression (64% neutropenia, 18% thrombocytopenia, 31% grade 3/4 anemia) [4]; 3rd-line therapy nivolumab, does not prolong overall survival [5]. Chemotherapy-related adverse events remain unresolved, highlighting the urgent need for the development of low toxicity and good druggability targeted therapies specifically designed for SCLC. Fortunately, researchers have made an exciting discovery in the search for a treatment for SCLC. They have found a new marine candidate drug named *N*-hydap that acts as a RoRγ antagonist, specifically targeting small cell tumor cells [6].

*N*-Hydap is a natural marine product that was found through the fermentation of a fungus called *Arthrinium arundinis* ZSDS1-F3, which is derived from sponges [7, 8]. It contains a chemical backbone known as a 4-hydroxy-2-pyridone alkaloid and is connected by a carbon bridge in the decalin moiety fraction. Approximately 2/3 of anti-cancer agents were derived from naturally occurring secondary metabolites [9]. Marine natural products, in particular, have shown great potential in fighting cancer with minimal harm to healthy cells, making them a promising and sustainable source of medicines [10]. Therefore, *N*-hydap has the potential to serve as a targeted drug candidate by inhibiting RoRγ activation [11], bringing hope for the advancement of more efficient and less toxic treatments for this aggressive type of lung cancer.

The properties of drug candidate ADME/T (drug absorption, distribution, metabolism, excretion, and toxicity) play a crucial role in the process of new drug development and throughout the development process [12]. The utilization of metabolic reaction systems (such as liver and kidney microsomes) in vitro to investigate drug metabolism in metabolic enzymes and analyze their metabolites, facilitates the prediction or extrapolation of drug biotransformation behavior in vivo [13]. In the preclinical pharmacological stage, the microsomal incubation test can be conducted using the cocktail in vitro probe method [14], in combination with high-throughput LC–MS rapid gradient elution technology, to assess the impact of drugs on in vitro liver CYP450 and UDP-glucuronosyltransferase enzymes activity [15]. This approach takes into account the pharmacological activity as well as the inhibition/induction of metabolic enzymes, which could lead to potential DDIs [16]. DDIs are a major concern in drug development, as they can lead to the termination of promising new treatments, removal of medications from the market, or stringent limitations on drug usage [17]. Etoposide, a widely used drug for SCLC treatment, has been found to possess repressive impacts on the function of CYP3A4 and CYP2C9 [17]. In clinical practice, special attention is also given to the potential drug interactions between cisplatin, or anti-PDL1 antibody atezolizumab when they are in combination therapy during cancer treatment [18, 19]. Patients with cancers are highly susceptible to DDIs due to the co-administration of anticancer drugs with other medications. The objective of learning the effect on DMEs of *N*-hydap is to identify novel compounds with potent pharmacological activity, while reducing the risk of metabolic interactions that can lead to severe DDIs and adverse reactions mediated by CYPs and UGTs inhibition or induction [20]. Therefore, it is significant to comprehensively study the metabolic characteristics and pharmacokinetic behavior of the marine drug candidate *N*-hydap in vitro and in vivo, which is conducive to early druggability evaluation, forming an integrated research system, and promotes the advancement of targeted drug research for SCLC.

## Results

### Determination of *N*-hydap and its metabolites in microsomes

**Fig. 1:**
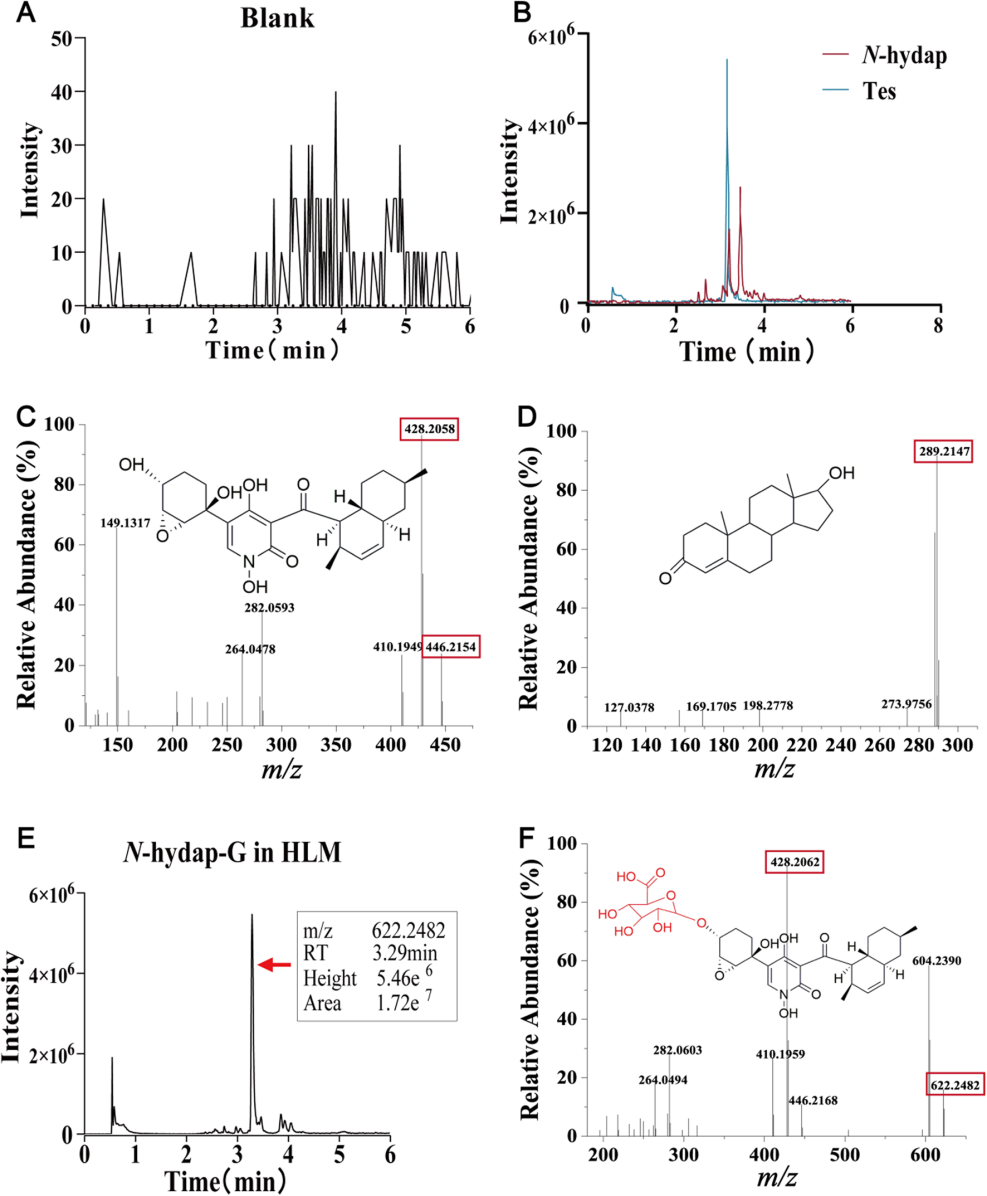
Structure of *N*-hydap and probable structure of *N*-hydap-G, HPLC chromatogram and the MS2 scan. **A** HPLC chromatogram of the blank. **B** HPLC chromatogram of *N*-hydap and Tes. **C** Structure and the MS2 scan of *N*-hydap. **D** Structure and the MS2 scan of Tes. **E** HPLC chromatogram of *N*-hydap-G in HLMs. **F** The probable structure and the MS2 scan of *N*-hydap-G

Simple LC–MS/MS analyses were conducted by LC-Orbitrap-Fusion-Tribrid-MS to investigate the metabolites of *N*-hydap in various microsomes. A blank control using 50% methanol–water was employed, and a robust LC–MS method was established. Figure 1A, B illustrate the chromatograms of the blank sample and the successful separation of *N*-hydap and the internal standard Tes, respectively. Subsequently, the UGT and CYP metabolites of *N*-hydap in various microsomes were identified.

**Fig. 2:**
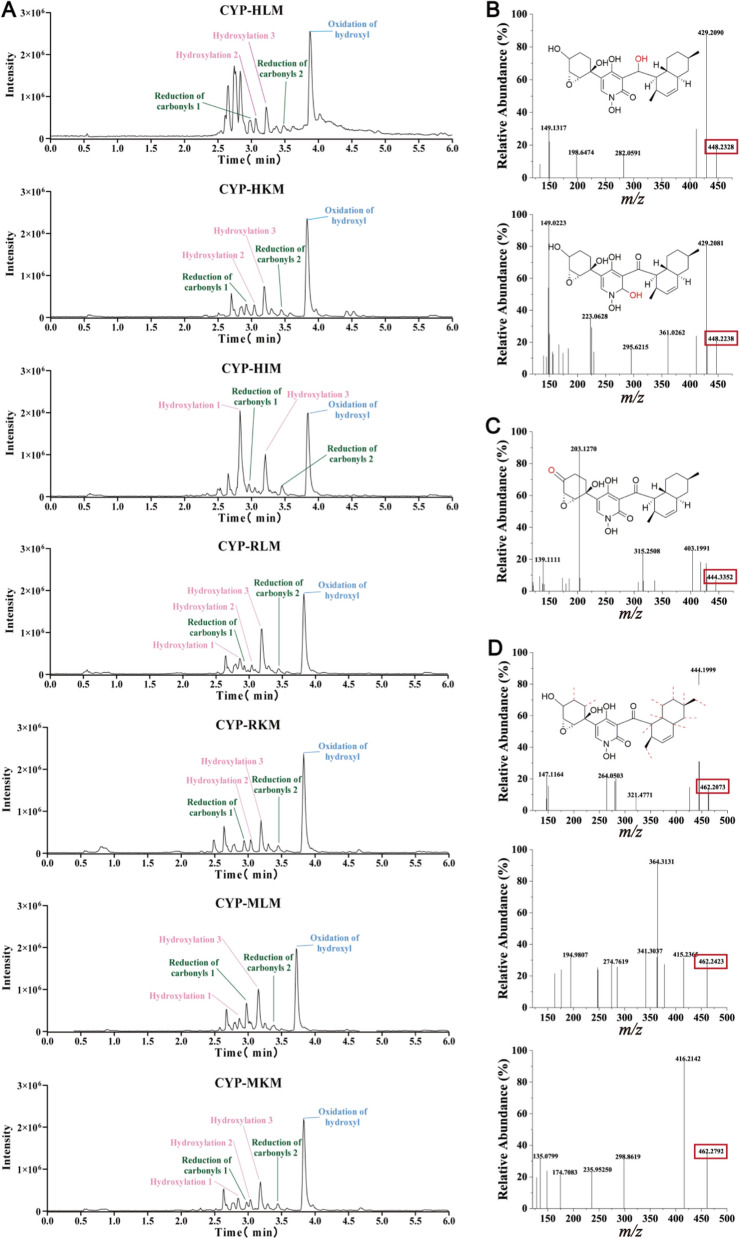
Probable structure, HPLC chromatogram and the MS2 scan of the CYP metabolites of *N*-hydap. **A** Total ion flow chromatograms of the CYP metabolites in various microsomes. The probable structure and the MS2 scan of reduction of carbonyls of *N*-hydap (**B**), oxidative metabolite of *N*-hydap (**C**) and hydroxylation of *N*-hydap (**D**)

*N*-Hydap (*m/z* 446.2154, calculated as C_24_H_31_NO_7_ + H^+^) and Tes (*m/z* 289.2147, calculated as C_19_H_28_O_2_ + H^+^) were identified (Fig. 1C, D). Then, it was found that in human liver microsomes (HLMs), *N*-hydap generated one glucuronide metabolite, *N*-hydap-G (*m/z* 622.2482, calculated as C_30_H_39_NO_13_ + H^+^) (Fig. 1E, F). As for CYP metabolites, there were one hydroxyl oxidation (*m/z* 443.3352, calculated as C_24_H_29_NO_7_ + H^+^), two carbonyl reductions (*m/z* 448.2328, 448.2238, calculated as C_24_H_33_NO_7_ + H^+^) and 2–3 hydroxylations in different microsomes (Fig. 2). *N*-Hydap exhibited similar metabolic patterns in human kidney microsomes (HKMs); human intestine microsomes (HIMs); rat liver microsomes (RLMs); rat kidney microsomes (RKMs); mouse liver microsomes (MLMs); mouse kidney microsomes (MKMs), producing the same glucuronide metabolite (Supplementary Fig. 2), hydroxyl oxidation and carbonyl reductions (Fig. 2). Regarding hydroxylation, we speculate that they are mono-hydroxylation products, resulting in 2–3 different products (*m/z* 462.2073, 462.2423, 462.2792, calculated as C_24_H_31_NO_8_ + H^+^) across various microsomes (Fig. 2D). In this research, the hydroxyl group on the six-membered ring containing the epoxy ring was found to be the most susceptible to biotransformation, with glucuronidation by UGTs being the most prominent pathway and it was prone to oxidation, resulting in the formation of carbonyls by CYPs. Additionally, CYP enzymes converted the two carbonyl groups in the *N*-hydap structure into hydroxyl groups. However, determining the precise hydroxylation position remains challenging.

### CYP- and UGT-mediated metabolism of *N*-hydap

**Fig. 3:**
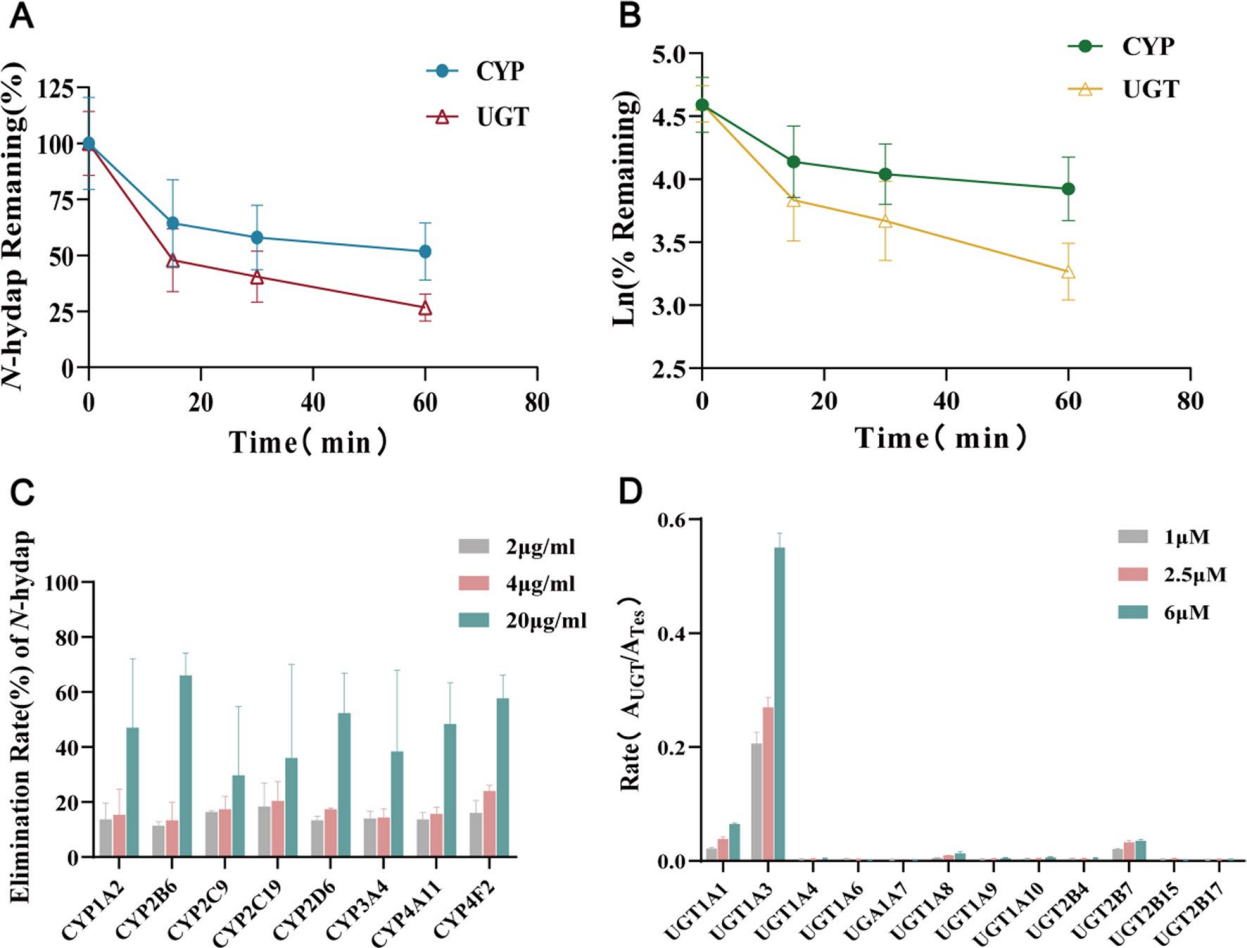
Comparison of CYP and UGT metabolisms of *N*-hydap, metabolism in recombinant CYPs and UGTs. **A** Comparison of *N*-hydap remaining between CYP and UGT reactions. **B** The natural logarithm of *N*-hydap remaining% against the incubation time. **C** *N*-hydap incubation with 8 human recombinant CYPs. **D** *N*-hydap incubation with 12 human recombinant UGTs (mean ± SD, n = 3)

In the CYP reaction using the same concentration of glucuronidation in HLMs, the remaining percentage of *N*-hydap was 64.43%, 58.02%, and 51.71% after 15, 30, and 60 min of reaction time, respectively. Conversely, in the glucuronidation reaction, the percentage of *N*-hydap remaining was 47.84%, 40.44%, and 26.72% after reaction times of 15, 30, and 60 min, respectively. After 1 h of microsomal incubation, only about 50% of the prototype drug remained in the CYP reaction, while less than 30% remained in the glucuronidation. It is worth noting that in the CYP reaction, the remaining percentage of *N*-hydap was higher compared to glucuronidation after incubation (Fig. 3A). Therefore, it is evident that glucuronidation was at least four times faster than CYP metabolism.

The natural logarithm of the residual rate of *N*-hydap against the incubation time was further graphed (Fig. 3B). *N*-Hydap demonstrated metabolic instability in HLMs, with a T_1/2_ of 178.98 min and a CL_int_ (in vivo) of 34.85 mL/min/kg during the CYP reaction. In the UGT reaction, the T_1/2_ and CL_int_ (in vivo) were 129.46 min and 48.18 mL/min/kg, respectively.

**Fig. 4:**
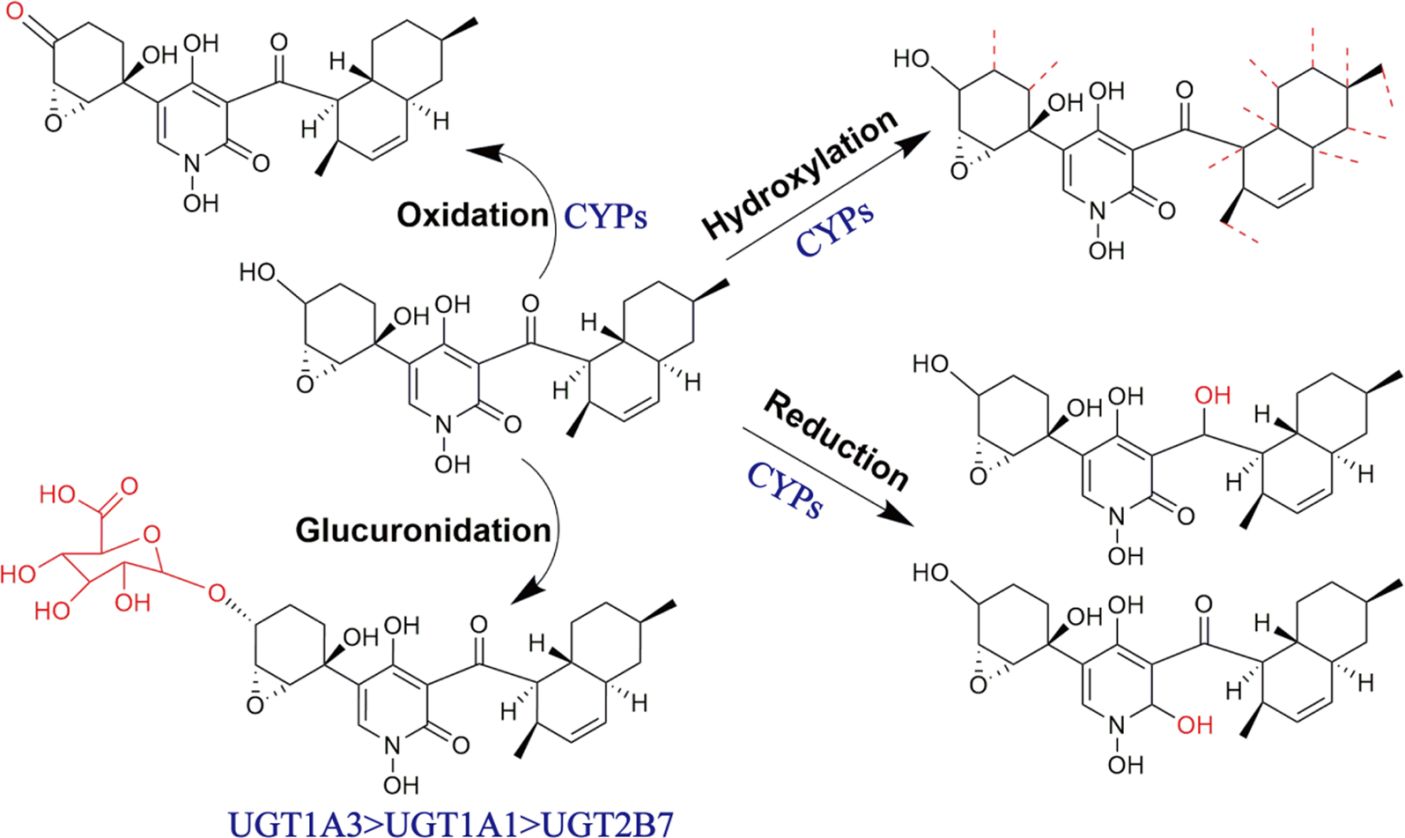
Summary of possible metabolites of *N*-hydap in microsomes

To identify the primary CYP or UGT isoforms responsible for metabolizing *N*-hydap, it was incubated with 8 recombinant human CYP enzymes and 12 UGT enzymes. The results revealed no significant difference in the metabolic elimination rate of *N*-hydap among the various recombinant human CYP enzymes, with the prototype elimination rate being less than 25% at enzyme concentrations of 2 and 4 μg/mL (Fig. 3C). UGT1A3 was the most significant contributor to the formation of *N*-hydap-G, accounting for over 80% of *N*-hydap glucuronidation, while UGT1A1 and UGT2B7 were secondary contributors (Fig. 3D). Conversely, only a negligible amount of *N*-hyda-G was detected after incubation with other UGT isoforms. Finally, a comprehensive overview of potential *N*-hydap metabolites and their corresponding metabolism pathway is presented in Fig. 4.

### Kinetics analysis of *N*-hydap glucuronidation

**Fig. 5:**
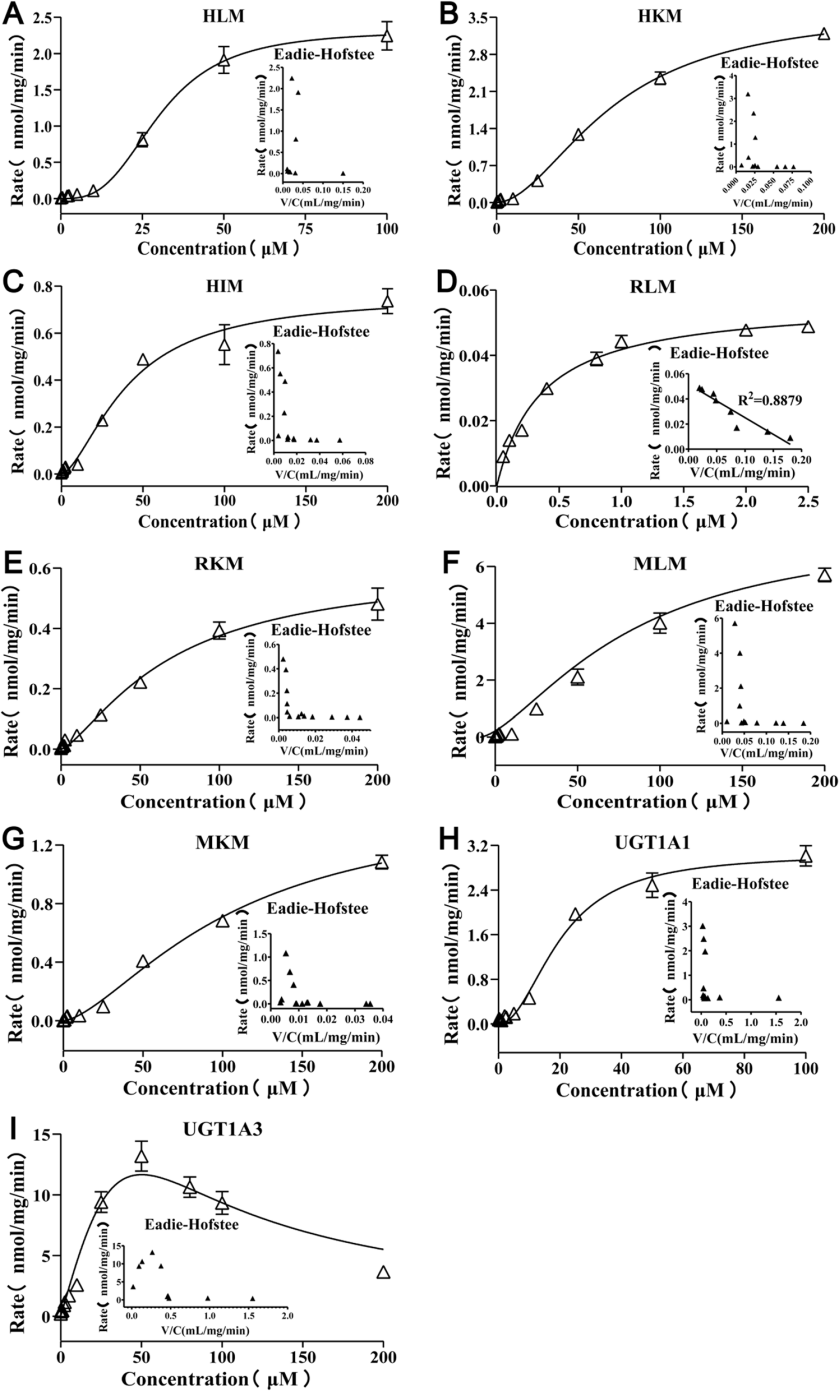
Kinetics profiles of *N*-hydap glucuronidation with Eadie–Hofstee plots by HLMs (**A**), HKMs (**B**), HIMs (**C**), RLMs (**D**), RKMs (**E**), MLMs (**F**), MKMs (**G**), UGT1A1 (**H**) and UGT1A3 (**I**), respectively (mean ± SD, n = 3)

We determined the glucuronidation rates of *N*-hydap by microsomes (HLMs, HKMs, HIMs, RLMs, RKMs, MLMs, and MKMs) and UGTs (UGT1A1 and UGT1A3) at various substrate concentrations. The enzyme kinetic model based on the enzymatic kinetic curve and the Eadie–Hofstee plot includes several profiles, such as the Michaelis–Menten hyperbolic kinetic profile, sigmoidal autoactivation profile, substrate inhibition profile, and biphasic kinetic profile [21]. Except for *N*-hydap glucuronidation in RLMs and UGT1A3, which followed Michaelis–Menten kinetics as evidenced by a linear Eadie–Hofstee plot (R^2^ = 0.8879) and substrate inhibition profile, respectively (Fig. 5D, I), the rest exhibited biphasic kinetic characteristics (Fig. 5A–C, E–H). All kinetic parameters of UGTs and microsomes’ catalyzed *N*-hydap glucuronidation were summarized in Table 1. Liver microsomes consistently displayed smaller K_m_ values and much higher CL_int_ values compared to corresponding kidney microsomes, irrespective of the species. Specifically, for K_m_, HLM < HKM (115.8 vs 239.6 μM), RLM < RKM (0.3569 vs 125.1 μM), and MLM < MKM (263.1 vs 404.8 μM). Similarly, for CL_int_, HLM > HKM (44.3 vs 30.1 μL/min/mg), RLM > RKM (158.9 vs 6.4 μL/min/mg), and MLM > MKM (51.2 vs 8.2 μL/min/mg). This suggests that liver microsomes had a greater affinity for *N*-hydap and a faster rate of catalyzing the conversion of *N*-hydap to glucuronide. Likewise, UGT1A3 exhibited smaller K_m_ values (10.68 vs 46.43 μM) and much higher CL_int_ values (942.9 vs 99.0 μL/min/mg) than UGT1A1. However, the V_max_ did not exhibit the same trend in different microsomes and UGTs.

**Table 1:** Enzyme kinetic parameters of *N*-hydap-G in microsomes (HLMs, HKMs, HIMs, RLMs, RKMs, MLMs and MKMs), and human recombinant UGTs (UGT1A1, UGT1A3)

Kinetic parameters	K_m_ (μM)	V_max_ (peak area ratio/min/mg)	CL_int_ (V_max_/K_m_, μL/min/mg)	R^2^
HLM	115.8 ± 46.46	5.126 ± 1.27	44.3	–
HKM	239.6 ± 37.96	7.210 ± 0.72	30.1	–
HIM	76.08 ± 11.29	1.020 ± 0.066	13.4	–
RLM	0.3569 ± 0.030	0.05670 ± 0.0014	158.9	0.8879
RKM	125.1 ± 15.78	0.8051 ± 0.052	6.4	–
MLM	263.1 ± 39.64	13.46 ± 1.32	51.2	–
MKM	404.8 ± 86.3	3.313 ± 0.51	8.2	–
UGT1A1	46.43 ± 8.00	4.596 ± 0.37	99.0	–
UGT1A3	10.68 ± 3.86	10.07 ± 0.98	942.9	–

Data are expressed as mean ± S.D (n = 3)

K_m_: Michaelis constant; V_max_: maximum reaction rate; CL_int_: intrinsic clearance

### Pharmacokinetic and distribution studies of *N*-hydap and *N*-hydap-G

**Fig. 6:**
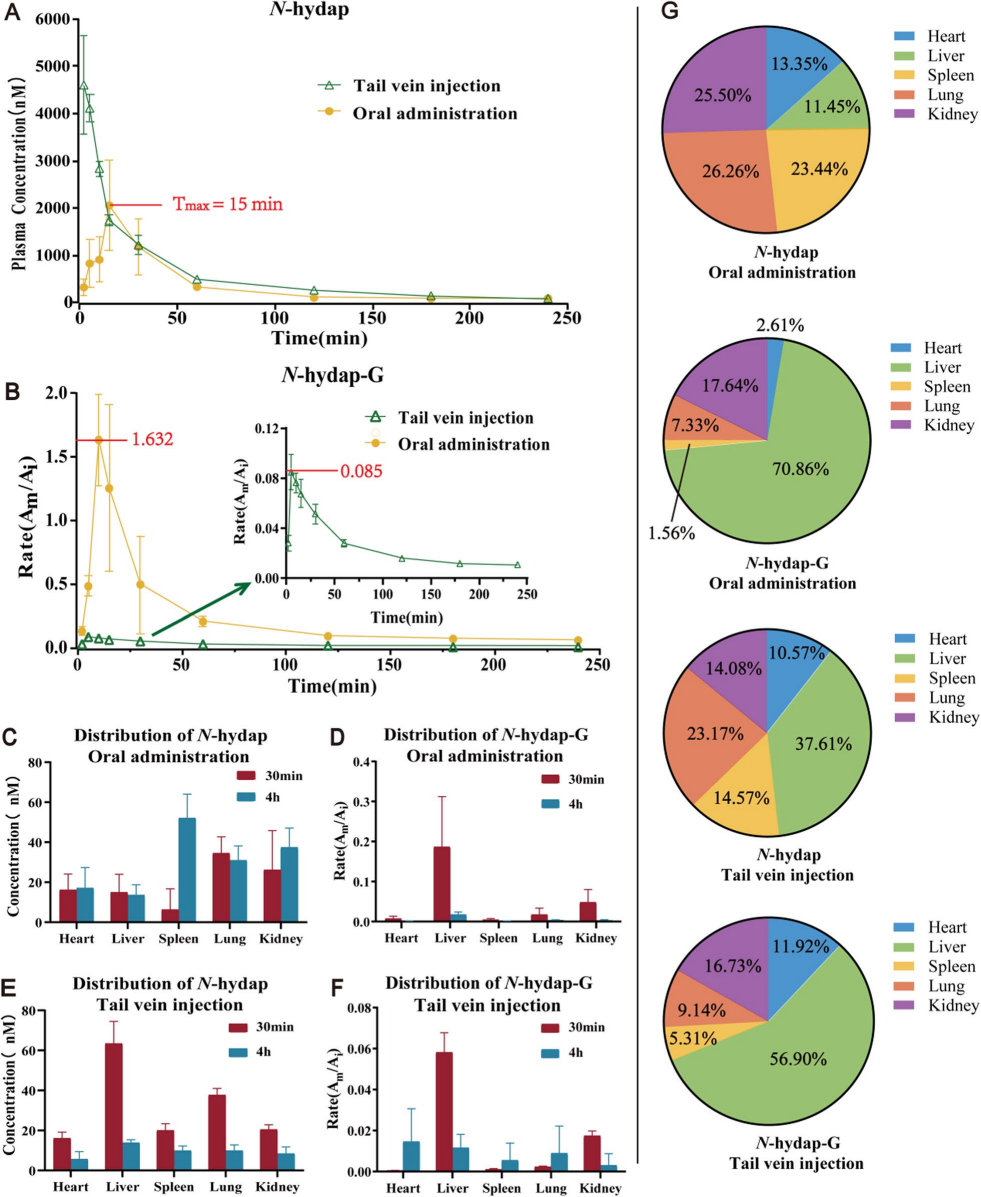
Plasma concentration and distribution of *N*-hydap and *N*-hydap-G after oral administration (5 mg/kg) and tail vein injection (2 mg/kg) in mice. **A** Plasma concentration of *N*-hydap after oral administration and tail vein injection. **B** Plasma concentration of *N*-hydap-G after oral administration and tail vein injection. **C**, **D** The distribution of *N*-hydap and *N*-hydap-G in organs after oral administration. **E**, **F** The distribution of *N*-hydap and *N*-hydap-G in organs after tail injection. **G** After gastric administration and intravenous injection, the total distribution ratio of *N*-hydap and *N*-hydap-G at 30 min and 4 h in various tissues (mean ± SD, n = 5)

In this study, we compared the pharmacokinetics and distribution patterns of *N*-hydap and *N*-hydap-G in mice following gastric administration and tail vein injection. A developed UPLC-MS/MS method was utilized to quantify plasma samples of *N*-hydap after oral administration (5 mg/kg) and tail vein injection (2 mg/kg, equivalent to oral administration of 5 mg/kg, theoretically). The plasma concentration–time profiles of *N*-hydap and *N*-hydap-G for both administration modes are depicted in Fig. 6A, B. The pharmacokinetic parameters were expressed as mean ± standard deviation, and all parameters are listed in Table 2.

**Table 2:** Pharmacokinetic parameters of *N*-hydap after oral administration (5 mg/kg) and tail vein injection (2 mg/kg) in mice (mean ± SD, n = 5)

Pharmacokinetic parameters	Oral administration	Tail vein injection
*C*_max_ (nM)	2058.112 ± 950.612	4788.630 ± 726.034***
*T*_max_ (min)	15.000 ± 0.000	3.000 ± 1.549
*AUC*_0−t_ (nM * min)	84764.772 ± 23252.634	141310.757 ± 7220.381***
t_1/2z_ (min)	57.568 ± 12.697	61.226 ± 14.185
*CLz*/*F* (L/min/kg)	0.134 ± 0.039	0.031 ± 0.001***
*F* (%)	24.0	

*C*_max_, maximum plasma concentration; *T*_max_, time to reach maximum plasma concentration; *AUC*_0–t_, area under the concentration–time curve from zero up to a definite time t; *t*_1/2z_, time for blood levels to drop by half; *CLz/F*, total clearance; *F*, oral bioavailability. Compared with oral administration, *** was p <0.001

Following oral administration, the plasma concentration–time curve exhibited a rapid initial increase followed by a sharp decline, with a half-life (T_1/2_) of 57.568 ± 12.697 min, until levels fell below the detection limit. Similarly, after *N*-hydap was administered through the tail vein, the plasma concentration decreased rapidly with a slower decrease thereafter, yielding a T_1/2_ of 61.226 ± 14.185 min. This indicates that *N*-hydap was rapidly eliminated in mice regardless of the administration route. Additionally, after oral administration, C_max_ and *AUC*_(0−t)_ showed a decrease, while *CL* exhibited an increase, indicating a faster elimination of *N*-hydap compared to tail vein injection, resulting in an oral bioavailability of 24.0%. We attribute this rapid clearance to the prompt conversion of *N*-hydap to *N*-hydap-G post-administration, as evident from the Plasma Rate (A_m_/A_i_) versus time profile (Fig. 6B). As shown in Fig. 6B, the highest concentration of *N*-hydap-G (A_m_/A_i_) in plasma after oral administration and tail vein injection were 1.632 and 0.085, respectively. Due to the doses of 5 mg/kg for oral administration and 2 mg/kg for intravenous injection, the maximum plasma concentration of *N*-hydap-G after oral gavage was 7.68 times that of intravenous injection (1.632/0.085/2.5 = 7.68).

After administering *N*-hydap to mice for 30 and 240 min, vital tissues and organs were extracted to assess the levels of *N*-hydap and *N*-hydap-G within them. The concentration of *N*-hydap and *N*-hydap-G (A_m_/A_i_) in organs after oral administering is depicted in Fig. 6C, D, and after tail vein injection is described in Fig. 6E, F. And then the total distribution ratio of *N*-hydap and *N*-hydap-G in various tissues is summarized in Fig. 6G. Following oral administration, the proportions of *N*-hydap distributed in the heart, liver, spleen, lungs, and kidneys were 13.35%, 11.45%, 23.44%, 26.26%, and 25.50%, respectively. The corresponding proportions of *N*-hydap-G were 2.61%, 70.86%, 1.56%, 7.33%, and 17.64%, respectively. After intravenous injection, the proportions of *N*-hydap distributed in the heart, liver, spleen, lungs, and kidneys were 10.57%, 37.61%, 14.57%, 23.17%, and 14.08%, respectively. The proportions of *N*-hydap-G were 11.92%, 56.90%, 5.31%, 9.14%, and 16.73%, respectively. Our findings revealed that *N*-hydap displayed a higher distribution in the liver, kidneys, and lungs, while *N*-hydap-G was predominantly concentrated in the liver and kidneys, two regions primarily responsible for drug metabolism.

### The toxicity of *N*-hydap

**Fig. 7:**
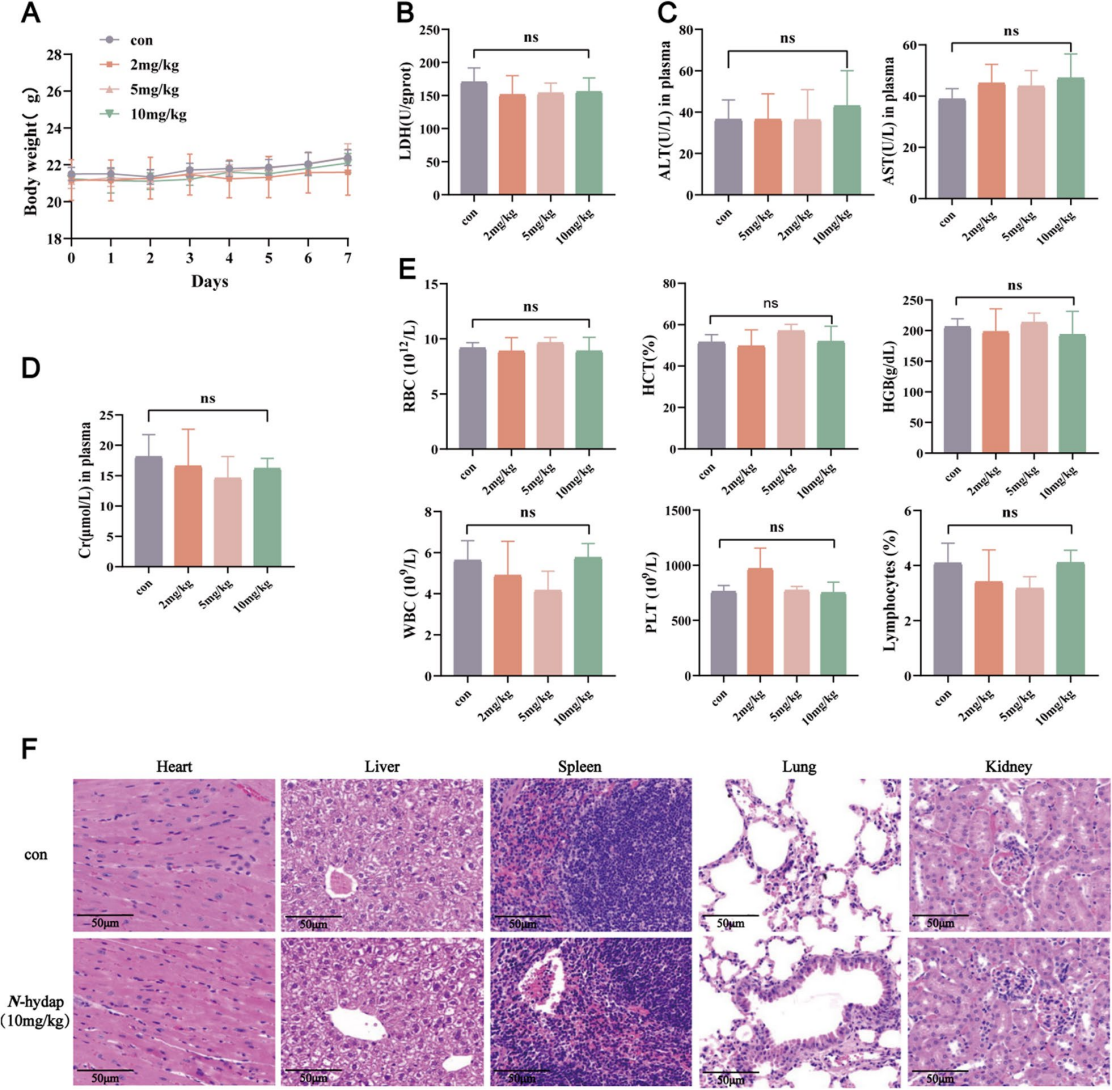
Normal wild-type mice were administered by *N*-hydap (2, 5, 10 mg/kg) gavage for 7 days. **A** Changes in body weight in mice. **B** Cardiac LDH level. **C**, **D** Plasma ALT, AST and Cr levels. **E** Blood routine indicators including RBC, HCT, HGB, WBC, PLT and Lymphocytes. **F** H&E staining of sections of primary organs, original magnification ×200 (mean ± S.D, n = 5–6/group). ns, no significance, were compared with control group. *LDH* lactate dehydrogenase, *ALT* alanine transaminase, *AST* aspartate aminotransferase, *Cr* creatinine, *RBC* red blood cell, *HCT* hematocrit, *HGB* hemoglobin, *WBC* white blood cell, *PLT* platelet

During the continuous gavage administration of *N*-hydap (2, 5, 10 mg/kg) to mice, their body weight was monitored daily and the mice’s activity and mental state were closely observed. No significant changes in body weight were observed among the different dosage groups after 7 days of continuous gavage. Moreover, the mice did not exhibit any abnormal behavior during the gavage process (Fig. 7A). To assess the extent of injury in the heart, liver, and kidneys, cardiac LDH, plasma ALT, AST, and Cr were measured as biochemical markers (Fig. 7B–D). Additionally, blood routines were examined, including RBC (10^12^/L), HCT (%), HGB (g/dL), WBC (10^9^/L), PLT (10^9^/L), and Lymphocytes (%), which were used as indicators to evaluate spleen toxicity (Fig. 7E). Encouragingly, the treated group did not show any significant differences compared to the control group in terms of the biochemical markers and blood routine indicators. Typically, a higher dose of a drug increases the likelihood of drug toxicity. To investigate this further, HE staining on tissue sections was performed from both the control group and the highest dose group (10 mg/kg). The important tissues and organs maintained their structural integrity, with cells arranged in an orderly manner and no evident infiltration of inflammatory cells (Fig. 7F). Based on the above results, it can be concluded that the continuous gavage administration of *N*-hydap at normal doses did not exhibit any toxicity in normal wild mice.

### Effects of *N*-hydap on mRNA and protein levels of DMEs

**Fig. 8:**
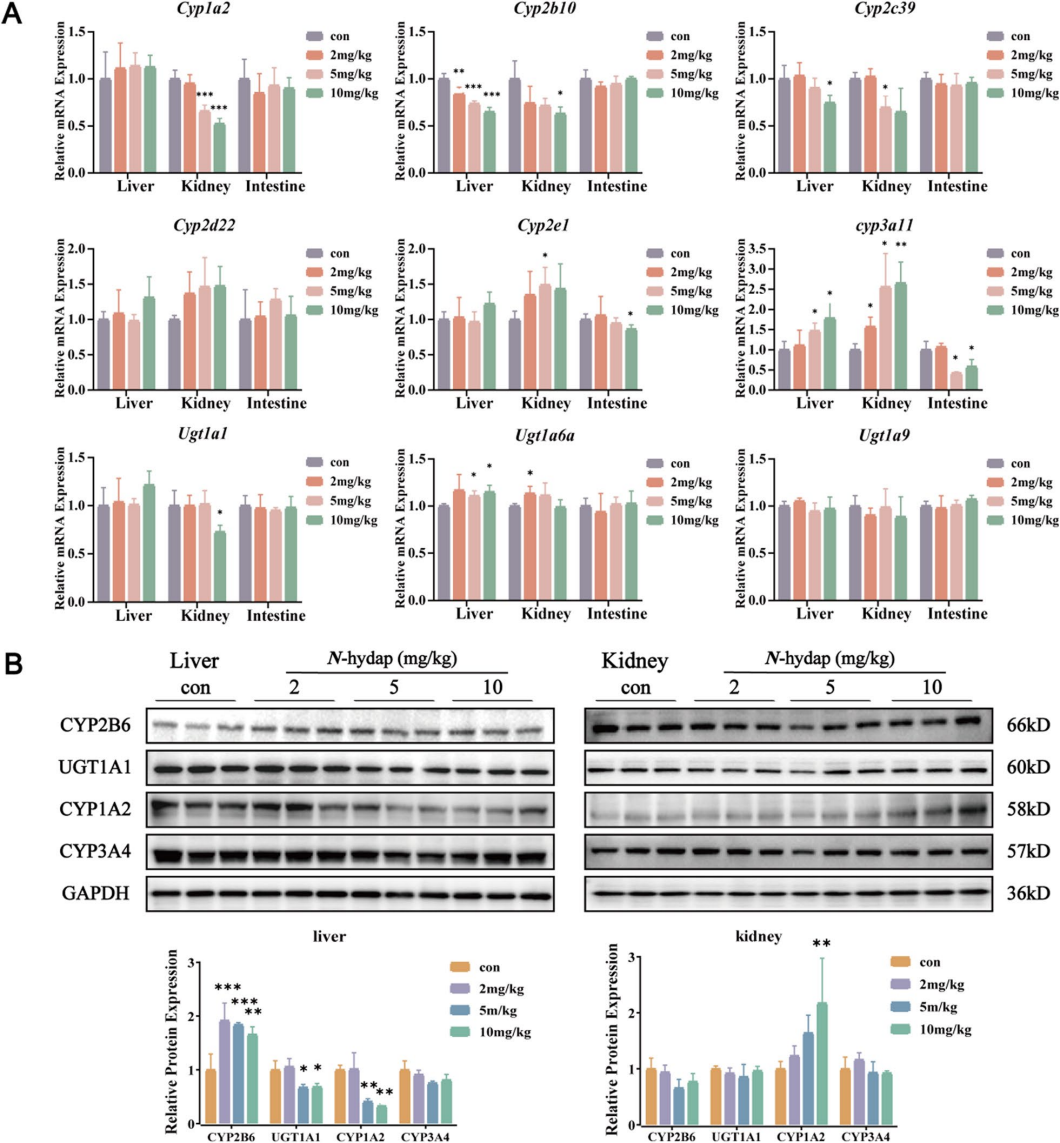
Effects of *N*-hydap on the mRNA (**A**, mean ± S.D, n = 5–6/group) and the protein levels (**B**, mean ± S.D, n = 3) of DMEs. *p < 0.05, **p < 0.01, and ***p < 0.001 compared with the control group

The mRNA and protein levels of CYPs and UGTs were examined following oral administration of *N*-hydap (2, 5, 10 mg/kg) to mice for 7 days. As depicted in Fig. 8A, *N*-hydap administration significantly decreased the mRNA levels of *Cyp1a2* in the kidneys by 48% and *Cyp2b10* in the liver by 35%. There was also a slight reduction in the mRNA levels of *Cyp2b10* in the kidneys, *Cyp2c39* in the liver and kidneys, *Cyp2e1* in the intestines, *Cyp3a11* in the intestines, and *Ugt1a1* in the kidneys. Conversely, exposure to *N*-hydap at the tested doses led to a modest increase in the mRNA levels of *Cyp2e1* in the kidney, *Cyp3a11* in the liver and kidneys (by 78%-160%), and *Ugt1a6a* in the liver and kidneys. No significant changes were observed in the mRNA levels of *Cyp2d22* and *Ugt1a9*.

Given that *N*-hydap had minimal impact on mRNA levels in the intestines overall, we focused on testing the protein levels in the liver and kidneys and explored the activity of liver microsomes and kidney microsomes in subsequent experiments. Western blot analysis was conducted to measure the protein levels of several crucial metabolic enzymes, including CYP1A2, CYP2B6, CYP3A4, and UGT1A1 (Fig. 8B). Following oral administration of *N*-hydap, in the liver, the protein levels of CYP2B6 were significantly increased by 92% and in contrast, the levels of UGT1A1 were slightly down-regulated and the levels of CYP1A2 were significantly decreased by 68%. Notably, in the kidney, *N*-hydap markedly up-regulated the levels of CYP1A2 by 117% at a dose of 10 mg/kg. However, there were no significant changes observed in the levels of CYP3A4 in either tissue after exposure to *N*-hydap. These findings indicate that the changes in protein expression and mRNA levels were not consistent.

### Effects of *N*-hydap on DMEs activity ex vivo

**Fig. 9:**
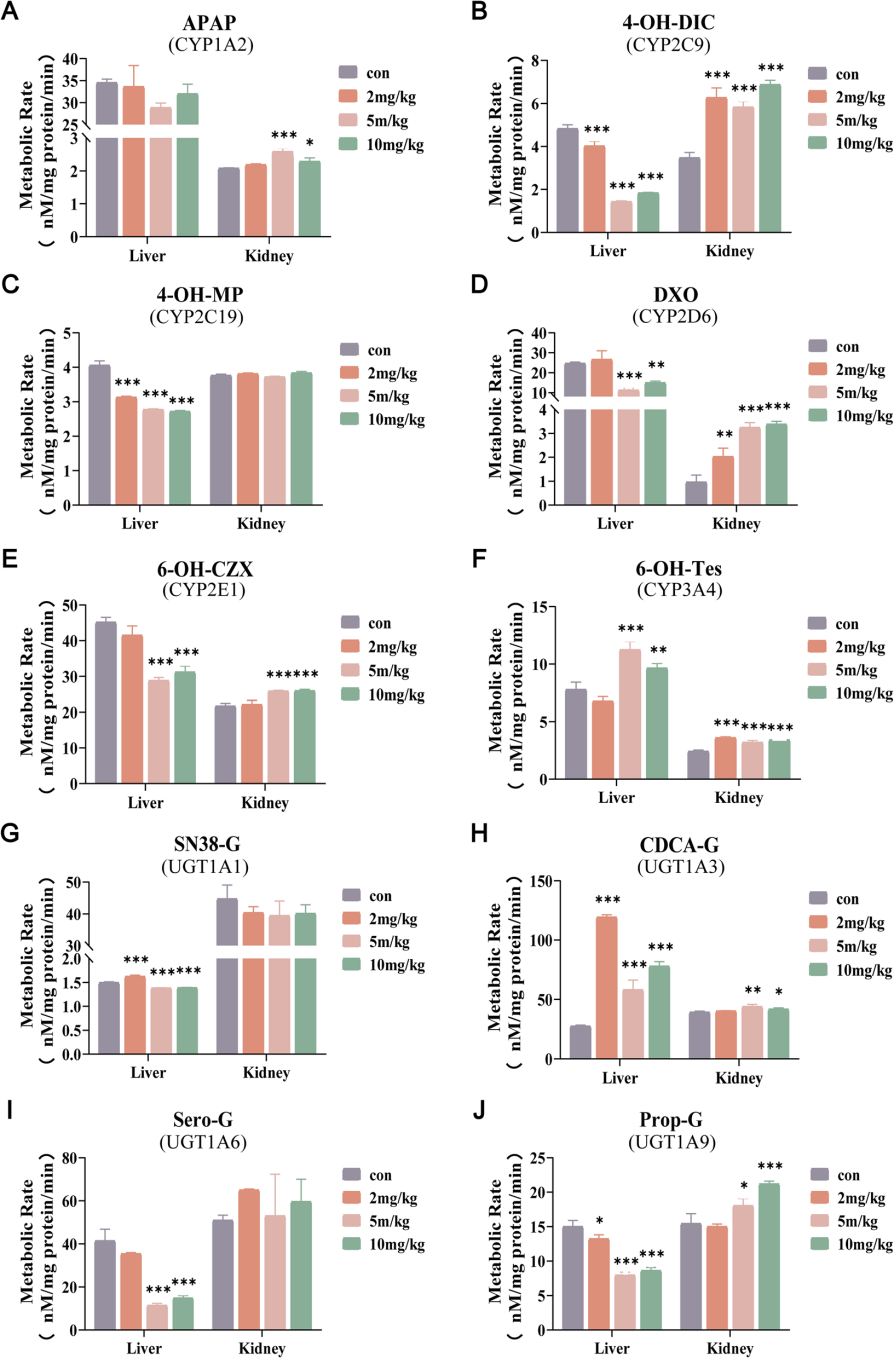
Formation rates of metabolites from substrate of DMEs in MLMs and MKMs made after oral administration of *N*-hydap. **A** CYP1A2. **B** CYP2C9. **C** CYP2C19. **D** CYP2D6. **E** CYP2E1. **F** CYP3A4. **G** UGT1A1. **H** UGT1A3. **I** UGT1A6. **J** UGT1A9. Data represent mean ± SD (n = 3). *p < 0.05, **p < 0.01, and ***p < 0.001 compared with the control group

The observed activity of key DMEs in MLMs and MKMs following a 7-day pretreatment with *N*-hydap (2, 5, or 10 mg kg^−1^ day^−1^, p.o.) or PBS (control group) in mice is depicted in Fig. 9. The optimization conditions for metabolites of DMEs’ substrates are presented in Supplementary Table 1 and their standard curves can be found in Supplementary Fig. 1D In liver microsomes, the formation rates of 4-OH-DIC, 4-OH-MP, DXO, Sero-G, and Prop-G were reduced by 70%, 33%, 54%, 72%, and 47%, respectively, compared to the control group. Conversely, these rates either increased or showed no substantial difference in kidney microsomes. Furthermore, the formation rates of APAP in MKMs, SN38-G in MLMs, and 6-OH-Tes and CDCA-G in both MLMs and MKMs were elevated by 12%-331%. In conclusion, the oral administration of *N*-hydap for 7 days exerted a notable influence on DMEs. Interestingly, while the activity of most enzymes in liver microsomes declined, it exhibited an opposite trend in renal microsomes. The effect of *N*-hydap on metabolic enzyme expression and activity is summarized in Table 3.

**Table 3:** The regulation of the main DMEs by *N*-hydap

DMEs	Regulatory level	Liver	Kidney
CYP1A2	Protein expression	–	↑
	Enzymatic activity	–	↑
CYP2C19	mNA expression	↓	↓
	Enzymatic activity	↓	–
CYP2D6	mNA expression	–	–
	Enzymatic activity	↓	↑
CYP2E1	mNA expression	–	↑
	Enzymatic activity	↓	↑
CYP3A4	mNA expression	↑	↑
	Enzymatic activity	↑	↑
UGT1A1	mNA expression	–	↓
	Enzymatic activity	↑	–
UGT1A6	mNA expression	↑	↑
	Enzymatic activity	↓	–
UGT1A9	mNA expression	–	–
	Enzymatic activity	↓	↑

↑: up-regulation; ↓: down-regulation; –: no change

## Discussion

Absorption is a key process by which a drug moves to circulatory system from the digestive tract, muscle and other site(s) of administration into body, influencing the blood drug levels as well as their pharmacological activities [22]. In our study, we found that the oral bioavailability may indeed be associated with absorption. Oral bioavailability refers to the relative amount of a drug that is absorbed into the systemic blood circulation after oral administration, with intravenous administration (generally considered to have a bioavailability of 100%) as a reference. The calculation of bioavailability involves the area under the concentration–time curve (AUC) for both oral and intravenous administration. For *N*-hydap given via gavage (5 mg/kg) and tail vein administration (2 mg/kg), the *AUC*_(0−t)_ values were 84764.772 ± 23252.634 vs 141310.757 ± 7220.381 nM*min, respectively. The calculated bioavailability was found to be 24.0%.

The literature suggests that the absorption of oral drugs may be affected by various factors, including drug solubility, intestinal absorption and first-pass effect, thereby affecting bioavailability. We believe the bioavailability after gavage (24.0%) may be plausibly explained by the metabolic profile of *N*-hydap that it was rapidly eliminated during both CYP- and UGT-mediated reactions, which is consistent with the concept of the first-pass effect. Compared to the CYP reaction, glucuronidation had a much higher metabolic rate. Additionally, the Rate (A_m_/A_i_)-Time curve of *N*-hydap-G demonstrated that the concentration of *N*-hydap-G was much higher after gavage compared to tail vein administration. Based on these findings, we conclude that the high rate of *N*-hydap conversion to *N*-hydap-G, first-pass elimination, resulted in an oral bioavailability of 24.0% for *N*-hydap.

Interestingly, *N*-hydap exhibited a relatively higher distribution in the lungs, accounting for 26.26% of the total distribution, second only to the liver and kidneys. This finding suggests that *N*-hydap not only has favorable oral bioavailability, but also demonstrates notable accumulation targeting the lungs, possessing strong activity against lung cancer. Despite being concentrated in the liver and kidneys, *N*-hydap did not exhibit toxicity in mice, indicating a high safety profile. Based on these results, we envisage that the development of a targeted lung formulation to enhance the bioavailability of *N*-hydap. Additionally, considering the significant exposure observed after intravenous injection, it can be inferred that *N*-hydap has the potential for intravenous administration.

With favorable oral bioavailability, *N*-hydap exhibited potent pharmacological activity against lung cancer and it was highly safe. Therefore, if *N*-hydap is further increased in oral bioavailability, it will be a good candidate for anti-SCLC. The drug metabolism and pharmacokinetics (DMPK)/DDIs profiles vary widely among small molecule drugs due to their structural and physicochemical characteristics [23]. Modifying the structure of *N*-hydap could potentially reduce its biotransformation rate by metabolic enzymes, thereby improving its oral bioavailability. In this study, the hydroxyl group on the six-membered ring containing the epoxy ring was identified as the most susceptible to biotransformation including glucuronidation and oxidation, leading to the formation of carbonyls. Modifying this hydroxyl group into more stable structures could potentially enhance the oral bioavailability of *N*-hydap. Similarly, CYP enzymes converted the two carbonyl groups into hydroxyl groups, providing an opportunity for modification of both fractions. Considering that the pharmacologically active structure of *N*-hydap is its parent nucleus, the 4-hydroxy-2-pyridone alkaloid [6], the potential modifications can be made to the outermost hydroxyl group and the carbonyl group on the lateral face.

Accurate prediction of pharmacokinetic properties is crucial for developing lead compounds with desirable drug-like characteristics. One key aspect is predicting hepatic clearance, which determines drug exposure and aids in predicting dosage, half-life, and bioavailability [24]. In vitro to in vivo extrapolation (IVIVE) is commonly used to predict hepatic clearance by employing microsomes or hepatocytes to determine in vitro intrinsic clearance, which is then scaled to estimate in vivo CL_int_ using physiologically relevant parameters such as microsomal protein content/hepatocellularity and liver weight [25]. In this study, in vitro, human microsomal incubation experiments were used to extrapolate in vivo liver clearance. The CL_int_ (in vivo) values for CYP response and UGT reaction were determined as 34.85 and 48.18 mL/min/kg, respectively, indicating a substantial hepatic clearance rate in vivo for *N*-hydap. These findings contribute to our understanding of *N*-hydap’s pharmacokinetic properties and can guide further research and development efforts.

Another major finding of this study is significant alterations in the mRNA expression and activity of several DMEs upon *N*-hydap exposure. Specifically, *N*-hydap strongly affected the expression and activity of CYP1A2, CYP2C19, CYP2D6, CYP2E1, CYP3A4, UGT1A1, UGT1A6, and UGT1A9. These enzymes play important roles in drug metabolism and are involved in DDIs. Furthermore, UGT1A3 was found to be the most crucial in the metabolism of *N*-hydap. After 7 days of *N*-hydap oral administration, the catalytic activity of UGT1A3 was significantly increased. UGT1A3 plays a vital role in conjugating exogenous compounds, such as drugs, chemotherapeutic agents, and carcinogens, as well as in the elimination of various endogenous compounds, including steroids, thyroid hormones, retinoic acids, and bilirubin [26]. Bile acids, particularly CDCA, are the main substrates for UGT1A3. The enhanced UGT1A3 activity in the liver and kidneys after *N*-hydap administration may result in accelerated metabolism and excretion of bile acids. This reduction in bile acid content can severely impair the absorption of lipids and lipophilic vitamins in the intestine, as well as alter blood cholesterol levels, leading to hypercholesterolemia and potentially resulting in atherosclerosis [27]. Moreover, CDCA activates the Farnesoid X receptor (FXR), which in turn affects other metabolic enzymes regulated by this transcription factor, thereby influencing liver and kidney metabolic function [28]. Imbalances in bile acid levels have also been reported to affect the enterohepatic circulation and disturb the balance of intestinal flora [29]. Thus, close monitoring is essential when administering *N*-hydap to avoid potential adverse DDIs and endogenous substance disorders.

Furthermore, our findings suggest that *N*-hydap downregulated the enzymatic activity of most DMEs in the liver, while it was interesting that the activity of several enzymes in the kidneys tended to be either opposite or unchanged. Literature reports suggest that drug metabolites can inhibit CYPs and UGTs [30]. Enzyme kinetic analysis of *N*-hydap glucuronidation revealed that liver microsomes exhibited a stronger affinity for *N*-hydap (lower K_m_ value) and a faster conversion rate of *N*-hydap to *N*-hydap-G (higher CL_int_ value) compared to renal microsomes in humans, rats and mice. We speculate that after 7 days of oral administration, *N*-hydap accumulated more in the liver, leading to increased levels of *N*-hydap-G, which may inhibit the activity of drug-metabolizing enzymes. This could explain the observed decrease in enzyme activity in the liver and the absence of significant changes or increases in the kidneys.

Many researchers now make use of online prediction platforms to assess the ADME/T properties of compounds, and highly recommend these platforms as invaluable tools for medicinal chemists during the drug discovery process, and they are freely accessible [31, 32]. According to the predicted results (Table 4), *N*-hydap was identified as a substrate of P-glycoprotein (P-gp) and an inhibitor of organic anion transporting polypeptides (OATP) 2B1. P-gp is responsible for exporting drugs from cells, thereby protecting the body against foreign substances, including anticancer agents [33]. OATP2B1 is an organic anion-transporting polypeptide, playing a crucial role in the absorption and disposition of various xenobiotics [34]. Hence, we hypothesize that the distinct pharmacokinetic profile observed following oral administration of *N*-hydap, compared to intravenous administration, may not solely be attributed to its rapid metabolism by metabolic enzymes. The involvement of transporters such as P-glycoprotein and OATP2B1 could also play a crucial role. Of course, further experimental investigations are needed to validate this hypothesis.

**Table 4:** Comparision of the ADME/T characteristics of *N*-hydap between prediction sites and the experimental results

Predicted content		Predicted results	Prediction sites	Experimental results
Absorption	OATP2B1 inhibitor	100% probability	Admet SAR	–
	P-gp substrate	Yes	ADMETlab	–
	P-gp substrate	Yes	PkCSM	–
	Bioavailability	55%	SwissADME	24.0%
Distribution	PPB	93.27%	ADMETlab	–
Metabolism	CYP3A4 substrate	71.26% probability	Admet SAR	Yes
	CYP2C9 substrate	60.44% probability	Admet SAR	Yes
	CYP1A2 substrate	Yes	ADMETlab	Yes
	CYP2D6 inhibitor	Yes	SwissADME	Inhibition in the liver
	CYP3A4 inhibitor	Yes	ADMETlab	No
	UGT catelyzed	80% probability	Admet SAR	Yes
	Phase I metabolism	Oxidation of hydroxyl, Reduction of carbonyls, Hydroxylation	BioTransformer 3.0	As predicted
	Phase II metabolism	Non	BioTransformer 3.0	Glucuronidation
Excretion	T_1/2_	Short Half-life	ADMETlab	< 1 h
	CL	High clearance	ADMETlab	High clearance
Toxicity	LD_50_	4000 mg/kg	ProTox-II	High security
	Hepatotoxicity	99% confidence	Xundrug	–
	Hepatotoxicity	Yes	PkCSM	–

“–” means no experiments were conducted

OATP2B1: organic anion transporting polypeptides; P-gp: P-glycoprotein; PPB: binding rate of plasma protein; T_1/2_: Half-life Time; CL: total clearance; LD_50_: median lethal dose

## Conclusion

In summary, this study successfully established a sensitive and reliable UPLC-MS method to investigate the metabolic profile and pharmacokinetic properties of *N*-hydap. Additionally, a fast and robust cocktail probe method was developed to assess the impact of *N*-hydap on the activity of DMEs. The findings revealed that N-hydap had an oral bioavailability of 24.0% due to its rapid metabolism by UGT enzymes, with UGT1A3 being the most significant contributor. Furthermore, *N*-hydap showed no toxicity and a high safety margin, with a higher distribution in the lungs, exhibiting potent inhibitory effects on SCLC cells. The insights gained from this study provide valuable information and guidance for the development of structurally modified analogs or derivatives of *N*-hydap as promising marine candidate drugs for the treatment of lung cancer.

## Experimental section

### Materials and reagents

Hydap (purity > 95%) is a 4-hydroxy-2-pyridone alkaloid as one of the prominent products extracted from the sponge-derived *Arthrinium arundinis* ZSDS1-F3. It was previously separated, purified, and identified by Junfeng Wang’s group [7, 8]. The fermented broth of *Arthrinium arundinis* was vacuum-condensed and then extracted with EtOAc to yield an EtOAc solution. The EtOAc extract was subjected to silica gel column chromatography and Sephadex LH-20 to obtain the initial purified subfractions. Subsequently, *N*-hydap was further purified via semipreparative HPLC using an ODS column eluting with 75% MeOH/H_2_O. Finally, the chemical structure of *N*-hydap was determined by NMR and HRMS spectra.

The recombinant human CYP isoforms (CYP1A2, CYP2B6, CYP2C9, CYP2C19, CYP2D6, CYP3A4, CYP4A11 and CYP4F2, 0.5 nmol) and the recombinant human UGT isoforms (UGT1A1, UGT1A3, UGT1A4, UGT1A6, UGT1A7, UGT1A8, UGT1A9, UGT1A10, UGT2B4, UGT2B7, UGT2B15 and UGT2B17, 5 mg/mL) were purchased from Cypex (Scotland, UK). The pooled HLMs, pooled HKMs, and pooled HIMs were bought from Corning Incorporated (Corning, NY, USA). The NADPH Regeneration System (including solution A and solution B) was bought from Promega (Madison, WI, USA). Uridine 5′-diphosphoglucuronic acid (UDPGA) was obtained from Sigma-Aldrich (St. Louis, MO, USA). d-Glucuric acid-1,4-monolactone-water, alamethicin, potassium phosphate dibasic (K_2_HPO_4_), potassium dihydrogen phosphate (KH_2_PO_4_), sucrose, magnesium chloride(MgCl_2_), EDTA dipotassium salt (EDTA·K_2_·2H_2_O), and testosterone (Tes, used as an internal standard, IS) were purchased from Aladdin (Shanghai, China). (2S,3S)-1,4-bis-sulfanylbutane-2,3-diol (DTT), Benzyl methylsulfonyl chloride (PMSF), and (±)-Verapamil hydrochloride (VER, used as an internal standard) were obtained from Shanghai Yuanye Bio-Technology Co., Ltd (Shanghai, China). Chlorzoxazone (CLP, used as an internal standard) was purchased from MeilunBio® (Liaoning, China). The study used primary antibodies against CYP1A2, CYP2B6, CYP3A4, and UGT1A1 produced by Abcam (Cambridge, MA, USA). Methanol and formic acid were HPLC grade. All analyses were performed in ultrapure water. Regarding the remaining chemicals and solvents, they were of analytical grade or higher quality and utilized as supplied.

### Animal

Animals were used with approval from Southern Medical University’s Ethics Committee in all cases. Male C57BL/6 mice weighing between 22 and 25 g as well as SD rats weighing between 200 and 250 g were obtained from Guangzhou Jinwei Biotechnology Co., Ltd (Guangdong, China). In a pathogen-free environment, the animals were provided with rodent pellets and sterile water, with a 12-h light–dark cycle.

### Preparation of RLMs, RKMs, MLMs, MKMs

In this study, the rats were executed by drawing blood from the abdominal aorta and mice were sacrificed with cervical dislocation before the liver and kidneys were excised. The liver and kidneys were removed, and washed with chilled cleansing solution (contains 8 mM KH_2_PO_4_, 5.6 mM EDTA·K_2_·2H_2_O, 1 mM DTT and 229.6 μM PMSF), homogenized in a tissue homogenate buffer with a pH of 7.4, (consisting of 1.8 μM KH_2_PO_4_, 8 mM K_2_HPO_4_, 250 mM sucrose, 1 mM EDTA·K_2_·2H_2_O and 229.6 μM PMSF). Subsequently, the homogenate was centrifuged at 10,400 rpm at 4 °C for 15 min, and the supernatant was then centrifuged at 35,000 rpm at 4 °C for 60 min. A 250 mM sucrose buffer was added to the pellet to reconstitute and the resuspension was stored at − 80 °C until use. Microsome protein concentrations were measured by Omni-Easy™ Instant BCA Protein Assay Kit.

### LC–MS/MS analysis

4000 Q TRAP mass spectrometer (AB SCIEX LLC, Redwood City, CA, USA) with an AB SCIEX electrospray ionization (ESI) source was connected to a Nexera X2 LC system consisting of a degasser, binary pump, autosampler, and thermostatic column compartment and operated with AB SCIEX MultiQuant version 3.0.2 software (AB SCIEX LLC, Redwood City, CA, USA). We performed a chromatography separation on a Phenomenex Kinetex XB-C18 column (100 × 2.10 mm, 2.6 μM) using solvents A (0.01% formic acid and 2 mM ammonium formate in water) and B (methanol) as the mobile phase, with gradient elution at 0.4 mL/min. In order to achieve optimal elution conditions, the following conditions must be met: 0–0.3 min, 10% B; 0.3–1.0 min, 10–90% B; 1.0–3.0 min, 90% B; 3.0–3.1 min, 90–10% B; 3.1–6.0 min, 10% B. Column oven temperature was kept at room temperature, and the injection volume was 5 μL. The mass spectrometer was in ESI modes that switch positive/negative ions, and ions were detected by multiple reaction monitoring (MRM) mode. An overview of the MRM transitions and the compound dependent parameters is shown in Supplementary Table 1. The operating conditions of the MS were optimized as follows: Curtain Gas (CUR), 30 psi; Collision Gas (CAD), medium; Ion Spray Voltage (IS), 5500 V; Temperature (TEM), 550 °C; Ion Source Gas1 (GS1), 55 psi; Ion Source Gas2 (GS2), 55 psi.

### Method validation

To verify the feasibility and reliability of the established method, studies on the linearity, accuracy, precision, and stability of *N*-hydap in potassium phosphate buffer (KPI, pH 7.4, were prepared by mixing KH_2_PO_4_ and K_2_HPO_4_ in corresponding proportions, and then adding NaOH and H_3_PO_4_ to pH 7.4), plasma, and tissue homogenate were carried out. The peak area ratios (analyte/IS) were plotted against the concentrations of the analytes prepared to obtain the calibration curves. Least squares linear regression analysis was used to determine the slopes, intercepts, and determination coefficients (R^2^) which should be 0.99 or above of calibration curves. The standard curve equations were determined as y = 0.0001512x + 0.04394 (R^2^ = 0.9980) for KPI, y = 0.001579x − 0.09233 (R^2^ = 0.9923) for plasma, and y = 0.002271x + 0.03542 (R^2^ = 0.9978) for tissue homogenate (Supplementary Fig. 1A–C).

To determine the accuracy and precision of the analytes within and between days, five samples were spiked at three quality control concentrations, either on the same day or on 3 consecutive days. Accuracy (%) was calculated by determining the percentage deviation from the theoretical concentration [(observed value of concentration)/(true value of concentration) × 100%]. Precision (%) was obtained by calculating the relative standard deviation (% RSD) for inter- and intra-day replicates. These precisions remained within the acceptable 15% limit and all measurements demonstrated satisfactory accuracy, with recovery percentages ranging from 85 to 115% of the reference value, as shown in Supplementary Table 2.

The stability of the KPI solution was examined after storage at 4 °C for 24 h and incubation at 37 °C for 4 h, which was conducted using the QC samples at three concentrations (five samples for each concentration). Stability in plasma and tissue homogenate was assessed by testing samples stored at both 4 °C and room temperature for 24 h. The results of the stability experiments indicated no significant degradation (Supplementary Table 3). In conclusion, the standard curves, inter- and intra-day precision, accuracy, and stability all met the required criteria for working with KPI solution, plasma, and tissue homogenate.

### Incubation of *N*-hydap with microsomes

Various microsomes including HLMs, HKMs, HIMs, RLMs, RKMs, MLMs and MKMs were involved in CYP and UGT reactions of *N*-hydap. A typical CYP incubation mixture included 0.5 mg/mL microsomes protein, 3.3 mM glucose-6-phosphate, 3.3 mM MgCl_2_, 0.4 U/mL glucose-6-phosphate dehydrogenase, and *N*-hydap (1, 2.5, or 10 μM) in the 50 mM potassium phosphate buffer (KPI, pH 7.4) [35]. The incubation mixture was preincubated at 37 °C for 5 min, and the reaction was started by adding NADP to form a NADPH-regenerating system. Simultaneously, the classic UGT reaction system consisted of 50 mM KPI, 5 mM MgCl_2_, 0.5 mg/mL microsomes, 0.125 mg/mL alamethicin, and 5.25 mg/mL saccharolactone. The reaction was initiated after supplying UDPGA. The CYP and UGT mixtures were incubated at 37 °C in a shaking water bath (80 rpm) for 1.5 h to enhance metabolite formation. To terminate the reaction, an ice-cold solution of Tes (50 ng/mL, dissolved in methanol and used as an internal standard) was added to half of the mixture volume. The mixture was vortexed for 30 s and then centrifuged at 13,000 rpm for 15 min to eliminate any precipitated protein. The resulting supernatant was transferred to autosampler vials, and 5 μL was injected into the LC–MS system. All reactions were conducted in triplicate.

### Assay for metabolic stability in HLMs

The identical concentration of HLMs was utilized for both the CYP reaction and UGT reaction. At predetermined time intervals of 0, 15, 30, and 60 min, 100 µL samples were taken from the reaction volumes. To halt the reaction, 50 µL of 50 ng/mL Tes was added. Samples were analyzed using LC–MS to compare the remaining level of *N*-hydap between the CYP reaction and UGT reaction. T_1__/2_ and CL_int_ (in vivo) (mL/min/kg) were calculated as reported previously [36]:

$${\text{T}}_{1/2}=0.693/\text{k},$$

where k represents the slope of the line derived from plotting the natural logarithm of the percentage (Ln %) of *N*-hydap remaining in the reaction mixture against the incubation time (minutes).

$$\begin{aligned}{\text{CL}}_{\operatorname{int}}\left( {{\text{in}}\;{\text{vivo}}} \right) & = \left( {0.693/{\text{in}}\;{\text{vitro}}\;{{\text{T}}_{1/2}}} \right) \times \left( {{\text{mL}}\;{\text{incubation}}/{\text{mg}}\;{\text{microsomes}}} \right) \\ &\quad \times \left( {45\;{\text{mg}}\;{\text{microsomes}}/{\text{g}}\;{\text{liver}}} \right) \times \left( {20\;{\text{g}}\;{\text{liver}}/{\text{kg}}\;{\text{body}}\;{\text{weight}}} \right).\end{aligned}$$

### Metabolism of *N*-hydap in recombinant human CYP and UGT enzymes

Incubation of *N*-hydap (2.5 μM) with eight recombinant human CYP enzymes (CYP1A2, CYP2B6, CYP2C9, CYP2C19, CYP2D6, CYP3A4, CYP4A11 and CYP4F2) at three enzyme concentrations (2, 4, and 20 μg/mL) was conducted following the same procedure as described above for microsomes. Subsequently, incubation with *N*-hydap (1, 2.5, and 6 μM) and twelve recombinant human UGT isoforms (UGT1A1, UGT1A3, UGT1A4, UGT1A6, UGT1A7, UGT1A8, UGT1A9, UGT1A10, UGT2B4, UGT2B7, UGT2B15 and UGT2B17) was performed, with the enzyme concentration used at 2 μg/mL. All reactions were carried out in triplicate.

### Kinetics of *N*-hydap glucuronidation in microsomes and UGTs

The substrate concentrations used for HLMs were 0.05, 0.5, 2, 2.5, 5, 10, 25, 50, and 100 μM. For HKMs, HIMs, RKMs, MLMs, and MKMs, the substrate concentrations used were 0.05, 0.1, 0.2, 0.4, 0.8, 1, 2, 2.5, 10, 25, 50, 100, and 200 μM. For RLMs, the substrate concentrations used were 0.05, 0.1, 0.2, 0.4, 0.8, 1, 2, and 2.5 μM. Lastly, for UGT1A1 and UGT1A3, the substrate concentrations used were 0.05, 0.25, 0.5, 1, 2, 2.5, 5, 10, 25, 50, 100, and 200 μM. Incubation time for all concentrations was 1 h. The rates of *N*-hydap-G by enzymes were expressed as nmol/mg/min, representing the amount of metabolites formed per minute per milligram of protein. Kinetic parameters were determined by analyzing the Eadie–Hofstee plots. Each incubation was performed in triplicate and repeated independently three times.

### Pharmacokinetics studies

Mice were fasted for 12 h with access to water before drug administration. *N*-Hydap was dissolved in a solution containing 50% methanol and water. Before administration, the drug was diluted with PBS to achieve concentrations of 5 mg/kg and 2 mg/kg for gastric gavage and tail vein administration, respectively. Blood samples (120 μL) were collected from the orbital venous plexus at 2, 5, 10, 15, 30, 60, 90, 120, 180, and 240 min after *N*-hydap administration. The blood samples were collected into heparinized micro-centrifuge tubes and immediately centrifuged at 8000 rpm for 5 min at 4 °C. Plasma samples were prepared by adding an internal standard (IS) solution, Tes to the samples, followed by vortexing. The mixtures were then centrifuged at 4 °C and 13,000 rpm for 15 min. The resulting supernatant was dried under vacuum for 3–4 h, and the dry powder was reconstituted with a solution of 50% methanol and water. Finally, the plasma samples were analyzed using LC–MS, following the previously described method.

### Distribution studies

The heart, liver, spleen, lungs and kidneys were removed from mice after administering a dose of 5 mg/kg through oral gavage and 2 mg/kg through tail vein injection at 30 min and 240 min, followed by homogenization with normal saline. The resulting homogenate was centrifuged at 4 °C, 13,000 rpm for 20 min, and the supernatant was then mixed with the internal standard by vortexing. The mixtures were centrifuged at 4 °C, 13,000 rpm for 15 min. The resulting supernatant was then subjected to vacuum drying for 3–4 h. Lastly, the dried powder was reconstituted with a mixture of 50% methanol and water, and then centrifuged for LC–MS testing.

### Drug metabolic enzyme activity in MLMs and MKMs

Mice were randomly divided into four groups, with eight animals in each group. The mice received oral pretreatment with *N*-hydap for 7 days at doses of 2, 5, and 10 mg/kg/day, which were considered as the low dose, medium dose, and high dose, respectively. Correspondingly, the control group received PBS. On the 8th day, the liver and kidneys were removed, and organs from eight mice per group were combined to prepare MLMs and MKMs using the detailed procedures above. Subsequently, we determined the activity of CYP and UGT enzymes by specific substrate metabolized with MLMs (0.5 mg/mL) and MKMs (0.2 mg/mL) in vitro. Specifically, the following selective substrates were used for the respective enzymes: PH (1 μM) for CYP1A2, DIC (1 μM) for CYP2C9, MP (90 μM) for CYP2C19, DM (1 μM) for CYP2D6, CZX (10 μM) for CYP2E1, Tes (1 μM) for CYP3A4, SN38 (1 μM) for UGT1A1, CACD (100 μM) for UGT1A3, Sero (20 μM) for UGT1A6, and Prop (1 μM) for UGT1A9. All substrates were dissolved in methanol or DMSO. The incubation procedure was carried out as mentioned above, and the reaction was terminated with VER (20 ng/mL) in ESI+ mode and CLP (200 ng/mL) in ESI− mode. In the ex vivo experiments, the activity of enzymes was measured by quantifying the production of various metabolites (nM/mg protein/min). The metabolites and their corresponding enzymes are as follows: APAP from PH, 4-OH-DIC from DIC, 4-OH-MP from MP, DXO from DM, 6-OH-CZX from CZX, 6-OH-Tes from Tes, SN38-G from SN38, CACD-G from CACD, Sero-G from Sero, Prop-G from Prop. The measurements were performed using multiple reaction monitoring (MRM) transitions, which are specific mass spectrometry transitions used for quantification. The optimized parameters for each metabolite, such as declustering potential and collision energy, were described in Supplementary Table 1. All experiments were conducted in triplicate, to ensure accuracy and reproducibility.

### Real-time PCR analysis

Total RNA from liver and kidney samples taken after 7 days of *N*-hydap gavage was extracted using the Animal Total RNA Isolation Kit (Foregene, Chengdu, Sichuan, China) following the manufacturer’s instructions. The concentrations of total RNA were determined by a nucleic acid concentration tester at 260/280 nm. A HiScript III RT SuperMix for qPCR (+ gDNA wiper) (Vazyme, Nanjing, Jiangsu, China) was used to measure the concentration of the synthesized cDNA. ChamQ SYBR qPCR Master Mix (Vazyme, Nanjing, Jiangsu, China) and detection were performed using a Light Cycler 480 II (ROCHE, Basel, Switzerland). Real-time PCR was used to analyze the relative mRNA levels of *Cyp1a2*, *Cyp2b10*, *Cyp2c39*, *Cyp2d22*, *Cyp2e1*, *Cyp3a11*, *Ugt1a1*, *Ugt1a6a* and *Ugt1a9* (human homologs: CYP1A2, CYP2B6, CYP2C19, CYP2D6, CYP2E1, CYP3A4, UGT1A1, UGT1A6, and UGT1A9) in the liver, kidneys, and intestines. The sequences of the primers used in the experiment are provided in Supplementary Table 4. The relative mRNA levels of the target gene were normalized against the levels of GAPDH mRNA.

### Western blot analysis

After 7 days of *N*-hydap gavage, the liver and kidney tissues were lysed using RIPA buffer supplemented with a 1% protease inhibitor cocktail. Protein concentrations were tested using a BCA estimation kit following the instructions provided by the manufacturer. Then the protein samples were mixed with 5× loading buffer and the mixture was denatured at 100 °C for 10 min. Equal amounts of protein (24 μg) were separated by 10% SDS-PAGE and subsequently transferred from the gel to the PVDF membrane. After blocking for 1–2 h with blocking fluid containing 0.1% Tween-20 (TBST), the corresponding primary antibodies were diluted according to the instructions and incubated with the membrane at 4 °C overnight. The membrane was washed before incubation with the corresponding secondary antibody at a dilution of 1:10,000 for 1–2 h at room temperature. Western blot signals were detected using an ECL chemiluminescence detection agent. The relative intensity of each protein band was scanned and quantified using Image J software.

### Data analysis

DAS 2.0 was used to calculate pharmacokinetic parameters using the standard non-compartmental method. All results were presented as mean ± standard deviation and significant differences were analyzed using One-way ANOVA by GraphPad Prism 8. Statistical significance was defined as p < 0.05.

### Supplementary Information

Supplementary Material 1.

## Data Availability

None.

## References

[1] Rudin CM, Brambilla E, Faivre-Finn C, Sage J. Small-cell lung cancer. Nat Rev Dis Prim. 2021;7(1):3. 33446664 10.1038/s41572-020-00235-0 PMC8177722

[2] Yang S, Zhang Z, Wang Q. Emerging therapies for small cell lung cancer. J Hematol Oncol. 2019;12(1):47. 31046803 10.1186/s13045-019-0736-3 PMC6498593

[3] Liu Q, Luo X, Yi L, Zeng X, Tan C. First-line chemo-immunotherapy for extensive-stage small-cell lung cancer: a united states-based cost-effectiveness analysis. Front Oncol. 2021;11: 699781. 34268124 10.3389/fonc.2021.699781 PMC8276096

[4] Eckardt JR, von Pawel J, Pujol JL, Papai Z, Quoix E, Ardizzoni A, Poulin R, Preston AJ, Dane G, Ross G. Phase III study of oral compared with intravenous topotecan as second-line therapy in small-cell lung cancer. J Clin Oncol. 2007;25(15):2086–92. 17513814 10.1200/JCO.2006.08.3998

[5] Owonikoko TK, Park K, Govindan R, Ready N, Reck M, Peters S, Dakhil SR, Navarro A, Rodriguez-Cid J, Schenker M, Lee JS, Gutierrez V, Percent I, Morgensztern D, Barrios CH, Greillier L, Baka S, Patel M, Lin WH, Selvaggi G, Baudelet C, Baden J, Pandya D, Doshi P, Kim HR. Nivolumab and ipilimumab as maintenance therapy in extensive-disease small-cell lung cancer: CheckMate 451. J Clin Oncol. 2021;39(12):1349–59. 33683919 10.1200/JCO.20.02212 PMC8078251

[6] Chen J, Hu Y, Zhang J, Wang Q, Wu X, Huang W, Wang Q, Cai G, Wang H, Ou T, Feng W, Liu P, Liu Y, Wang J, Huang J, Wang J. Therapeutic targeting RORgamma with natural product *N*-hydroxyapiosporamide for small cell lung cancer by reprogramming neuroendocrine fate. Pharmacol Res. 2022;178: 106160. 35259480 10.1016/j.phrs.2022.106160

[7] Han J, Liu C, Li L, Zhou H, Liu L, Bao L, Chen Q, Song F, Zhang L, Li E, Liu L, Pei Y, Jin C, Xue Y, Yin W, Ma Y, Liu H. Decalin-containing tetramic acids and 4-hydroxy-2-pyridones with antimicrobial and cytotoxic activity from the fungus *Coniochaeta cephalothecoides* collected in Tibetan Plateau (Medog). J Org Chem. 2017;82(21):11474–86. 29019245 10.1021/acs.joc.7b02010

[8] Wang J, Wei X, Qin X, Lin X, Zhou X, Liao S, Yang B, Liu J, Tu Z, Liu Y. Arthpyrones A-C, pyridone alkaloids from a sponge-derived fungus *Arthrinium arundinis* ZSDS1-F3. Org Lett. 2015;17(3):656–9. 25606827 10.1021/ol503646c

[9] Yang Y, Liu Q, Shi X, Zheng Q, Chen L, Sun Y. Advances in plant-derived natural products for antitumor immunotherapy. Arch Pharm Res. 2021;44(11):987–1011. 34751930 10.1007/s12272-021-01355-1

[10] Khalifa SAM, Elias N, Farag MA, Chen L, Saeed A, Hegazy MF, Moustafa MS, Abd El-Wahed A, Al-Mousawi SM, Musharraf SG, Chang FR, Iwasaki A, Suenaga K, Alajlani M, Goransson U, El-Seedi HR. Marine natural products: a source of novel anticancer drugs. Mar Drugs. 2019;17(9):491. 31443597 10.3390/md17090491 PMC6780632

[11] Zheng J, Wang J, Wang Q, Zou H, Wang H, Zhang Z, Chen J, Wang Q, Wang P, Zhao Y, Lu J, Zhang X, Xiang S, Wang H, Lei J, Chen HW, Liu P, Liu Y, Han F, Wang J. Targeting castration-resistant prostate cancer with a novel RORgamma antagonist elaiophylin. Acta Pharm Sin B. 2020;10(12):2313–22. 33354503 10.1016/j.apsb.2020.07.001 PMC7745055

[12] Ge G, Zhu M. Preface for special issue on new analytical techniques and methods in drug metabolism and pharmacokinetics. J Pharm Anal. 2020;10(3):iii–iv. 32612875 10.1016/j.jpha.2020.05.015 PMC7322737

[13] Saunders LJ, Fitzsimmons PN, Nichols JW, Gobas F. In vitro–in vivo extrapolation of hepatic and gastrointestinal biotransformation rates of hydrophobic chemicals in rainbow trout. Aquat Toxicol. 2020;228: 105629. 33002683 10.1016/j.aquatox.2020.105629 PMC7962060

[14] Sharma SS, Sharma S, Bureik M. Screening of the whole human cytochrome P450 complement (CYPome) with enzyme bag cocktails. J Pharm Anal. 2020;10(3):271–6. 32612874 10.1016/j.jpha.2020.05.003 PMC7322738

[15] Henderson GL, Harkey MR, Gershwin ME, Hackman RM, Stern JS, Stresser DM. Effects of ginseng components on c-DNA-expressed cytochrome P450 enzyme catalytic activity. Life Sci. 1999;65(15):PL209–14. 10574228 10.1016/S0024-3205(99)00407-5

[16] Wang JJ, Guo JJ, Zhan J, Bu HZ, Lin JH. An in-vitro cocktail assay for assessing compound-mediated inhibition of six major cytochrome P450 enzymes. J Pharm Anal. 2014;4(4):270–8. 29403890 10.1016/j.jpha.2014.01.001 PMC5761213

[17] Song JH, Sun DX, Chen B, Ji DH, Pu J, Xu J, Tian FD, Guo L. Inhibition of CYP3A4 and CYP2C9 by podophyllotoxin: implication for clinical drug-drug interactions. J Biosci. 2011;36(5):879–85. 22116286 10.1007/s12038-011-9143-9

[18] Zhang Y, Kuchimanchi M, Zhu M, Doshi S, Hoang T, Kasichayanula S. Assessment of pharmacokinetic interaction between rilotumumab and epirubicin, cisplatin and capecitabine (ECX) in a phase 3 study in gastric cancer. Br J Clin Pharmacol. 2017;83(5):1048–55. 27966237 10.1111/bcp.13179 PMC5401968

[19] Yang SY, Yi JM, Chun J, Park S, Bui TT, Yun HY, Chae JW, Jeong MK. Evaluation of the potential herb–drug interaction between Bojungikki-tang and PD-L1 immunotherapy in a syngeneic mouse model. Front Pharmacol. 2023;14:1181263. 37274110 10.3389/fphar.2023.1181263 PMC10232755

[20] Xie H, Wu J, Liu D, Liu M, Zhang H, Huang S, Xiong Y, Xia C. In vitro inhibition of UGT1A3, UGT1A4 by ursolic acid and oleanolic acid and drug-drug interaction risk prediction. Xenobiotica. 2017;47(9):785–92. 27600106 10.1080/00498254.2016.1234087

[21] Seibert E, Tracy TS. Different enzyme kinetic models. Methods Mol Biol. 2014;1113:23–35. 24523107 10.1007/978-1-62703-758-7_3

[22] Nakanishi T, Tamai I. Interaction of drug or food with drug transporters in intestine and liver. Curr Drug Metab. 2015;16(9):753–64. 26630906 10.2174/138920021609151201113537

[23] Takakusa H, Iwazaki N, Nishikawa M, Yoshida T, Obika S, Inoue T. Drug metabolism and pharmacokinetics of antisense oligonucleotide therapeutics: typical profiles, evaluation approaches, and points to consider compared with small molecule drugs. Nucleic Acid Ther. 2023;33(2):83–94. 36735616 10.1089/nat.2022.0054 PMC10066781

[24] Tess D, Chang GC, Keefer C, Carlo A, Jones R, Di L. In vitro–in vivo extrapolation and scaling factors for clearance of human and preclinical species with liver microsomes and hepatocytes. AAPS J. 2023;25(3):40. 37052732 10.1208/s12248-023-00800-x

[25] Sodhi JK, Benet LZ. Successful and unsuccessful prediction of human hepatic clearance for lead optimization. J Med Chem. 2021;64(7):3546–59. 33765384 10.1021/acs.jmedchem.0c01930 PMC8504179

[26] Fang ZZ, He RR, Cao YF, Tanaka N, Jiang C, Krausz KW, Qi Y, Dong PP, Ai CZ, Sun XY, Hong M, Ge GB, Gonzalez FJ, Ma XC, Sun HZ. A model of in vitro UDP-glucuronosyltransferase inhibition by bile acids predicts possible metabolic disorders. J Lipid Res. 2013;54(12):3334–44. 24115227 10.1194/jlr.M040519 PMC3826681

[27] Thomas C, Pellicciari R, Pruzanski M, Auwerx J, Schoonjans K. Targeting bile-acid signalling for metabolic diseases. Nat Rev Drug Discov. 2008;7(8):678–93. 18670431 10.1038/nrd2619

[28] Meech R, Hu DG, McKinnon RA, Mubarokah SN, Haines AZ, Nair PC, Rowland A, Mackenzie PI. The UDP-glycosyltransferase (UGT) superfamily: new members, new functions, and novel paradigms. Physiol Rev. 2019;99(2):1153–222. 30724669 10.1152/physrev.00058.2017

[29] Ticho AL, Malhotra P, Dudeja PK, Gill RK, Alrefai WA. Intestinal absorption of bile acids in health and disease. Compr Physiol. 2019;10(1):21–56. 31853951 10.1002/cphy.c190007 PMC7171925

[30] Liu S, Hou L, Li C, Zhao Y, Yao X, Zhang X, Tian X. Contributions of UDP-glucuronosyltransferases to human hepatic and intestinal metabolism of ticagrelor and inhibition of UGTs and cytochrome P450 enzymes by ticagrelor and its glucuronidated metabolite. Front Pharmacol. 2021;12: 761814. 34721047 10.3389/fphar.2021.761814 PMC8552062

[31] Dong J, Wang NN, Yao ZJ, Zhang L, Cheng Y, Ouyang D, Lu AP, Cao DS. ADMETlab: a platform for systematic ADMET evaluation based on a comprehensively collected ADMET database. J Cheminform. 2018;10(1):29. 29943074 10.1186/s13321-018-0283-x PMC6020094

[32] Tian H, Ketkar R, Tao P. ADMETboost: a web server for accurate ADMET prediction. J Mol Model. 2022;28(12):408. 36454321 10.1007/s00894-022-05373-8 PMC9903341

[33] Waghray D, Zhang Q. Inhibit or evade multidrug resistance P-glycoprotein in cancer treatment. J Med Chem. 2018;61(12):5108–21. 29251920 10.1021/acs.jmedchem.7b01457 PMC6281405

[34] Chen M, Hu S, Li Y, Gibson AA, Fu Q, Baker SD, Sparreboom A. Role of Oatp2b1 in drug absorption and drug–drug interactions. Drug Metab Dispos. 2020;48(5):419–25. 32114507 10.1124/dmd.119.090316 PMC7180048

[35] Liu W, Shi J, Zhu L, Dong L, Luo F, Zhao M, Wang Y, Hu M, Lu L, Liu Z. Reductive metabolism of oxymatrine is catalyzed by microsomal CYP3A4. Drug Des Dev Ther. 2015;9:5771–83. 10.2147/DDDT.S92276 PMC4636097 26586934

[36] Jeong H, Lee J, Kim S, Yeo YY, So H, Wu H, Song YS, Jang CY, Kim HD, Kim MJ, Chang M. Hepatic metabolism of sakuranetin and its modulating effects on cytochrome P450s and UDP-glucuronosyltransferases. Molecules. 2018;23(7):1542. 29949932 10.3390/molecules23071542 PMC6100415

