# Correction: Metabolism characterization and toxicity of *N*-hydap, a marine candidate drug for lung cancer therapy by LC–MS method

**DOI:** 10.1007/s13659-024-00464-w

**Published:** 2024-08-02

**Authors:** Jindi Lu, Weimin Liang, Yiwei Hu, Xi Zhang, Ping Yu, Meiqun Cai, Danni Xie, Qiong Zhou, Xuefeng Zhou, Yonghong Liu, Junfeng Wang, Jiayin Guo, Lan Tang

**Affiliations:** 1 NMPA Key Laboratory for Research and Evaluation of Drug Metabolism, Guangdong Provincial Key Laboratory of New Drug Screening, Guangdong-Hong Kong-Macao Joint Laboratory for New Drug Screening, Southern Medical University Hospital of Integrated Traditional Chinese and Western Medicine, School of Pharmaceutical Sciences, Southern Medical University, Guangzhou, 510515 China; 2 CAS Key Laboratory of Tropical Marine Bio-Resources and Ecology/Guangdong Key Laboratory of Marine Materia Medica, South China Sea Institute of Oceanology, Chinese Academy of Sciences, Guangzhou, 510301 China

**Correction: Natural Products and Bioprospecting (2024) 14:33** 10.1007/s13659-024-00455-x

Following publication of the original article [1], the authors reported that the original version of this article unfortunately contained mistakes.

- Page 1, section “Abstract”, the originally published texts were: Despite low bioavailability (0.024%), *N*-hydap exhibited a higher distribution in the lungs (26.26%), accounting for its efficacy against SCLC.
  The correct texts should read: With a favorable bioavailability of 24.0%, *N*-hydap exhibited a higher distribution in the lungs (26.26%), accounting for its efficacy against SCLC.
- Page 2, the presentation of Graphical Abstract “Low bioavailability (0.024%)” was incorrect.
  The originally published Graphical Abstract was:
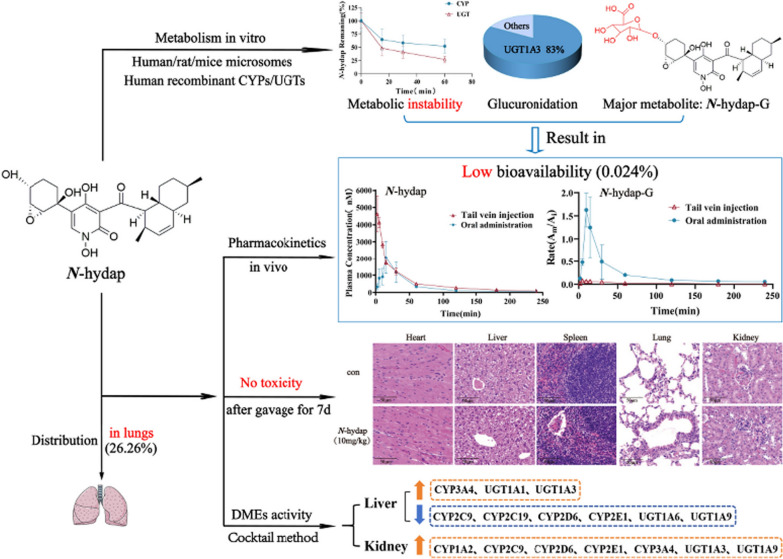
  The corrected detail is Bioavailability (24.0%) and the corrected Graphical Abstract is shown below:
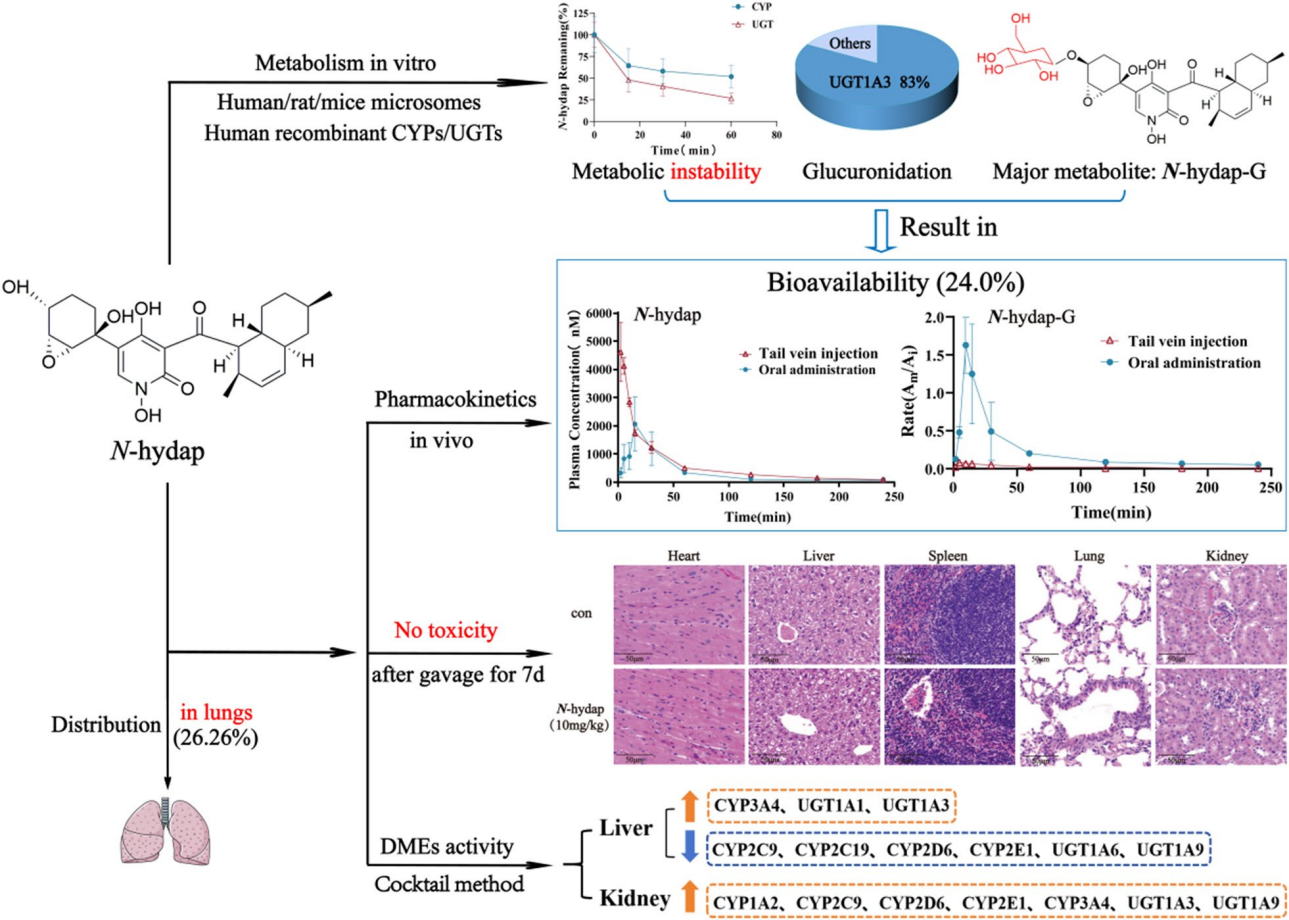
- Page 7, section “2.4 Pharmacokinetic and distribution studies of *N*‑hydap”, second paragraph, the originally published texts were: Additionally, after oral administration, C_max_ and AUC_(0−t)_ showed a decrease, while CL exhibited an increase, indicating a faster elimination of *N*-hydap compared to tail vein injection, resulting in an oral bioavailability of only 0.024%.
  The corrected texts should read: Additionally, after oral administration, *C*_max_ and *AUC*_(0−t)_ showed a decrease, while *CL* exhibited an increase, indicating a faster elimination of *N*-hydap compared to tail vein injection, resulting in an oral bioavailability of 24.0%.
- Page 11, the presentation of Table 2 was incorrect.
  The originally published Table 2 was:
  **Table 2:** Pharmacokinetic parameters of *N*-hydap after oral administration (5 mg/kg) and tail vein injection (2 mg/kg) in mice (mean ± SD, n = 5)

Pharmacokinetic parameters	Oral administration	Tail vein injection
C_max_ (μg/L)	0.916 ± 0.423	2131.946 ± 339.265
T_max_ (min)	15 ± 0	3 ± 1.549
AUC_0−t_ (μg/L * min)	37.738 ± 10.352	62,912.962 ± 3214.586
t1/2z (min)	57.568 ± 12.697	61.226 ± 14.185
CLz/F (L/min/kg)	143.162 ± 39.558	0.031 ± 0.001
F (%)	0.024	

C_max_: maximum plasma concentration; T_max_: time to reach maximum plasma concentration; AUC_0−t_: area under the concentration–time curve from zero up to a definite time t; t1/2z: time for blood levels to drop by half; CLz/F: total clearance; F: oral bioavailability
  The corrected Table 2 is given below:

**Table 2:** Pharmacokinetic parameters of *N*-hydap after oral administration (5 mg/kg) and tail vein injection (2 mg/kg) in mice (mean ± SD, n = 5)

Pharmacokinetic parameters	Oral administration	Tail vein injection
*C*_max_ (nM)	2058.112 ± 950.612	4788.630 ± 726.034***
*T*_max_ (min)	15.000 ± 0.000	3.000 ± 1.549
*AUC*_0−t_ (nM * min)	84,764.772 ± 23,252.634	141,310.757 ± 7220.381***
t_1/2z_ (min)	57.568 ± 12.697	61.226 ± 14.185
*CL*z/*F* (L/min/kg)	0.134 ± 0.039	0.031 ± 0.001***
*F* (%)	24.0	

*C*_max_: maximum plasma concentration; *T*_max_: time to reach maximum plasma concentration; *AUC*_0–t_: area under the concentration–time curve from zero up to a definite time t; *t*_1/2z_: time for blood levels to drop by half; *CL*z/*F*: total clearance; *F*: oral bioavailability

Compared with oral administration, *** was p < 0.001
- Page 11, section “Discussion”, first paragraph, the original texts were: In our study, we found that the low oral bioavailability may indeed be associated with poor absorption.
  The corrected texts should read: In our study, we found that the oral bioavailability may indeed be associated with absorption.
- Page 11, section “Discussion”, first paragraph, the originally published texts were: For *N*-hydap given via gavage (5 mg/kg) and tail vein administration (2 mg/kg), the AUC(0 − t) values were 37.738 ± 10.352 μg/L * min and 62,912.962 ± 3214.586 μg/L * min, respectively. The calculated bioavailability was found to be 0.024%, indicating that the low bioavailability of *N*-hydap is indeed caused by poor absorption.
  The corrected texts should read: For *N*-hydap given via gavage (5 mg/kg) and tail vein administration (2 mg/kg), the *AUC*_(0−t)_ values were 84764.772 ± 23252.634 vs 141310.757 ± 7220.381 nM * min, respectively. The calculated bioavailability was found to be 24.0%.
- Page 11, section “Discussion”, second paragraph, the originally published texts were: the original texts were: We believe the low bioavailability after gavage (0.024%) may be plausibly explained by the metabolic profile of *N*-hydap that it was rapidly eliminated during both CYP- and UGT-mediated reactions, which is consistent with the concept of the first-pass effect.
  The corrected texts should read: We believe the bioavailability after gavage (24.0%) may be plausibly explained by the metabolic profile of *N*-hydap that it was rapidly eliminated during both CYP- and UGT-mediated reactions, which is consistent with the concept of the first-pass effect.
- Page 11, section “Discussion”, second paragraph, the originally published texts were: Based on these findings, we conclude that the high rate of *N*-hydap conversion to *N*-hydap-G resulted in the extremely low oral bioavailability of *N*-hydap.
  The corrected texts should read: Based on these findings, we conclude that the high rate of *N*-hydap conversion to *N*-hydap-G, first-pass elimination, resulted in an oral bioavailability of 24.0% for *N*-hydap.
- Page 11, section “Discussion”, third paragraph, the originally published texts were: This finding suggests that although *N*-hydap has low bioavailability, it possesses strong activity against lung cancer.
  The corrected texts should read: This finding suggests that *N*-hydap not only has favorable oral bioavailability, but also demonstrates notable accumulation targeting the lungs, possessing strong activity against lung cancer.
- Page 11, section “Discussion”, fourth paragraph, the originally published texts were: Despite its low oral bioavailability, *N*-hydap exhibited potent pharmacological activity against lung cancer and *N*-hydap was highly safe. Therefore, if *N*-hydap is increased in oral bioavailability, it will be a good candidate for anti-SCLC.
  The corrected texts should read: With favorable oral bioavailability, *N*-hydap exhibited potent pharmacological activity against lung cancer and it was highly safe. Therefore, if *N*-hydap is further increased in oral bioavailability, it will be a good candidate for anti-SCLC.
- Page 15, section “Conclusion”, the originally published texts were: The findings revealed that *N*-hydap had an extremely low oral bioavailability due to its rapid metabolism by UGT enzymes, with UGT1A3 being the most significant contributor.
  The corrected texts should read: The findings revealed that *N*-hydap had an oral bioavailability of 24.0% due to its rapid metabolism by UGT enzymes, with UGT1A3 being the most significant contributor.
- Page 16, the content “0.024%” of the fifth row, fifth column of the Table 4 was incorrect. The corrected content is 24.0%.
  The original article [1] has been updated.
