# Supplementary material for: Metabolism characterization and toxicity of N-hydap, a marine candidate drug for lung cancer therapy by LC–MS method
Source: Nat Prod Bioprospect. 2024 May 21;14(1):33. doi: 10.1007/s13659-024-00455-x (PMC11109052; doi:10.1007/s13659-024-00455-x)
Supplement: Supplementary file 1 — Supplementary Material 1. [file 13659_2024_455_MOESM1_ESM.docx]

**Metabolism characterization and toxicity of *N*-hydap, a marine candidate drug for lung cancer therapy by LC-MS**

**Supplementary materials**


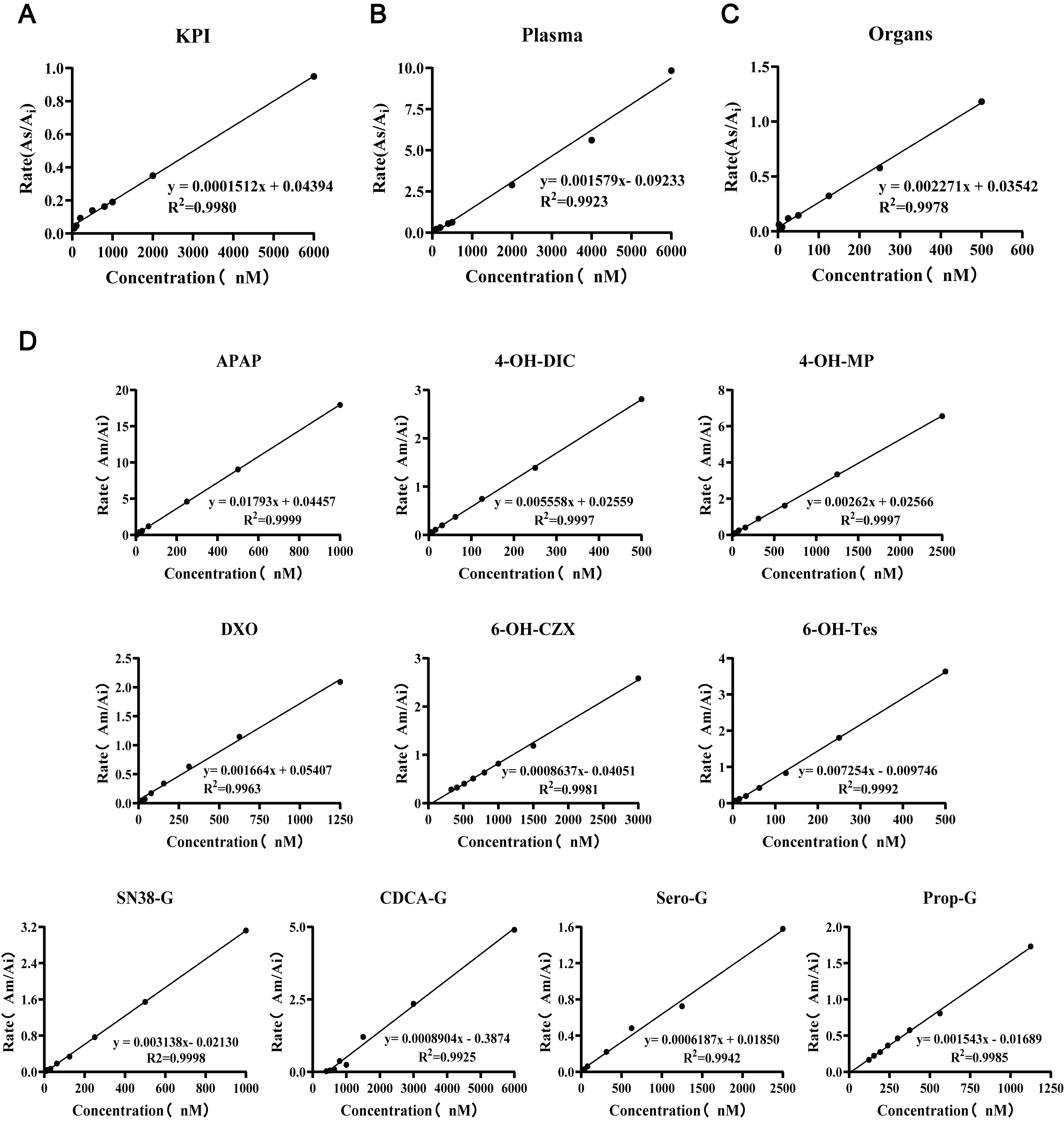


**Fig.1.** Standard curves of *N*-hydap in KPI (**A**), plasma (**B**), tissue homogenate (**C**) and the metabolites of DMEs' substrates (**D**).

KPI, potassium phosphate buffer.


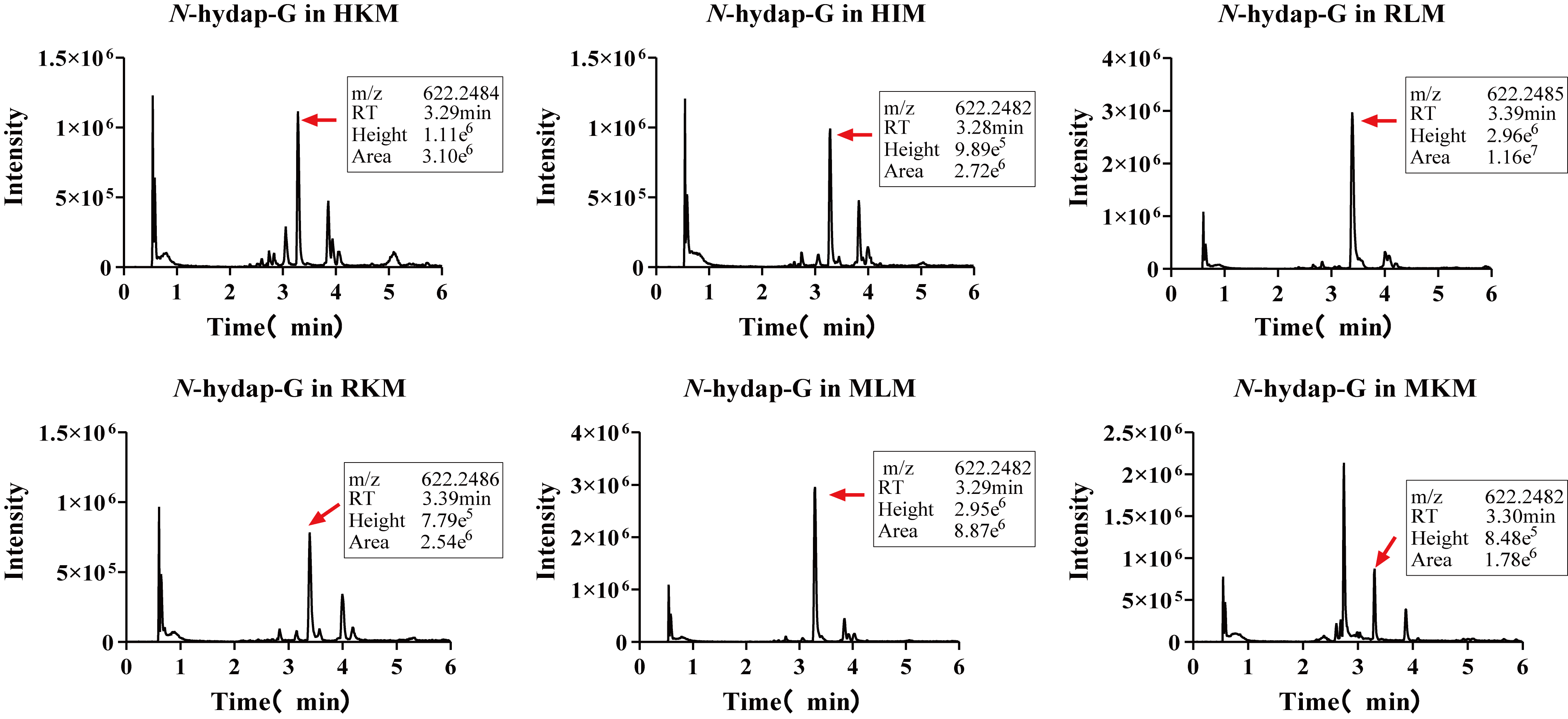


**Fig.2.** UPLC chromatogram of *N*-hydap-G in HKMs, HIMs, RLMs, RKMs, MLMs and MKMs.

**Table 1** Optimum conditions for ionization and fragmentation of *N*-hydap and metabolites of DMEs probe substrates and their ISs.

| **Ion mode** | **Analyte** | **Precursor ion and Product ion (*m/z*)** | **DP(V)** | **CE(V)** |
| --- | --- | --- | --- | --- |
| ESI+ | Tes(IS) | 289.4/97.2 | 43 | 96 |
|  | 1. hydap | 446.2/428.4 | 20 | 58 |
|  | APAP | 152.1/110.1 | 56 | 28 |
|  | 4-OH-DIC | 312.2/266.1 | 49 | 23 |
|  | 4-OH-MP | 235.3/150.3 | 90 | 35 |
|  | DXO | 258.2/157.1 | 56 | 28 |
|  | 6-OH-Tes | 305.4/269.2 | 62 | 25 |
|  | SN38-G | 569.5/393.5 | 106 | 43 |
|  | Sero-G | 353.2/160.4 | 86 | 48 |
|  | VER(IS) | 454.8/165.3 | 87 | 50 |
| ESI- | 6-OH-CLZ | 183.8/119.7 | -48 | -34 |
|  | CDCA-G | 567.6/391.1 | -68 | -57 |
|  | Prpo-G | 353.3/177.2 | -60 | -45 |
|  | CLP(IS) | 274.9/189.9 | -58 | -33 |

Notes: DP, declustering potential; CE, collision energy.

DMEs: drug metabolism enzymes; IS: internal standard.

**Table 2** Intra-day（n = 5） and inter-day (n = 3) precision and accuracy of *N*-hydap in KPI, plasma and tissue homogenate.

|  |  | **Intra-day** | | **Inter-day** | |
| --- | --- | --- | --- | --- | --- |
|  | Concentration（nM） | Precision  (RSD, %) | Accuracy  (Bias, %) | Precision  (RSD, %) | Accuracy  (Bias, %) |
| KPI | 200 | 12.54 | 99.75 | 2.57 | 98.45 |
|  | 600 | 4.58 | 102.33 | 2.29 | 102.43 |
|  | 2500 | 5.04 | 97.72 | 1.55 | 97.52 |
| Plasma | 250 | 6.84 | 104.36 | 3.82 | 99.96 |
|  | 600 | 5.73 | 103.20 | 4.91 | 108.68 |
|  | 2500 | 3.09 | 108.76 | 2.66 | 112.16 |
| Tissue homogenate | 40 | 11.74 | 97.08 | 8.42 | 107.50 |
|  | 150 | 9.36 | 101.60 | 3.11 | 98.60 |
|  | 500 | 2.64 | 99.72 | 2.62 | 99.88 |

**Table 3** The stability of *N*-hydap in KPI, plasma and tissue homogenate (n = 5).

|  |  | 37℃ for 4h | | 4℃ for 24h | |
| --- | --- | --- | --- | --- | --- |
|  | Concentration（nM） | Precision  (RSD, %) | Accuracy  (Bias, %) | Precision  (RSD, %) | Accuracy  (Bias, %) |
| KPI | 200 | 13.24 | 87.75 | 10.22 | 113.85 |
|  | 600 | 7.28 | 85.75 | 12.25 | 105.48 |
|  | 2500 | 3.72 | 97.32 | 2.48 | 104.40 |
|  |  | room temperature for 24h | | 4℃ for 24h | |
|  | Concentration（nM） | Precision  (RSD, %) | Accuracy  (Bias, %) | Precision  (RSD, %) | Accuracy  (Bias, %) |
| Plasma | 200 | 6.55 | 101.9 | 9.45 | 113.9 |
|  | 600 | 11.35 | 105.23 | 10.43 | 87.98 |
|  | 2500 | 3.47 | 108.04 | 1.85 | 91.36 |
| Tissue homogenate | 60 | 14.03 | 103.17 | 14.58 | 102.62 |
|  | 150 | 3.24 | 99.13 | 9.04 | 103.40 |
|  | 500 | 6.33 | 93.38 | 3.21 | 105.16 |

**Table 4** Gene-specific polymerase chain reaction primers.

| Oligo Name | Sequence（5'-3'） | |
| --- | --- | --- |
| *Cyp1a2* | Forward | AGTACATCTCCTTAGCCCCAG |
|  | Reverse | GGTCCGGGTGGATTCTTCAG |
| *Cyp2b10* | Forward | TGCTGTCGTTGAGCCAACC |
|  | Reverse | CCACTAAACATTGGGCTTCCT |
| *Cyp2c39* | Forward | GAGGAAGCATTCCAATGGTAGAA |
|  | Reverse | TGTGAAGCGCCTAATCTCTTTC |
| *Cyp2d22* | Forward | TGGTTGTACTAAATGGGCTGAC |
|  | Reverse | GCTAGGACTATACCTTGAGAGCG |
| *Cyp2e1* | Forward | TGCGGAGGTTTTCCCTAAGTA |
|  | Reverse | TGTGCCTCTCTTTGGATGCG |
| *Cyp3a11* | Forward | GTGCTCCTAGCAATCAGCTT |
|  | Reverse | CAGTGCCTAAAAATGGCAGAGG |
| *Ugt1a1* | Forward | CACTGGCTGAGTATGCTTGG |
|  | Reverse | CTTCTGGAATGGCACAGGGAA |
| *Ugt1a6a* | Forward | GTTTCTCTTCCTAGTGCTTTGGG |
|  | Reverse | CCTCGTTCACTGAGATGTTCTAC |
| *Ugt1a9* | Forward | TCTCGCTCCCATCAGTGATCTT |
|  | Reverse | TGGTTCCACACTCTCTCCTTG |
| *Gapdh* | Forward | AGGTCGGTGTGAACGGATTTG |
|  | Reverse | TGTAGACCATGTAGTTGAGGTCA |
